# Indigenous and local knowledge inclusion in forest fauna research: A systematic review in the tropics

**DOI:** 10.1007/s13280-026-02378-y

**Published:** 2026-04-24

**Authors:** Carlos Alberto Hernández-Vélez, Guy Jackson, Torsten Krause

**Affiliations:** 1 Lund University Center for Sustainability Studies, Lund University, Box 170, 22100 Lund, Sweden; 2 Department of Social Sciences, Northumbria University, Room 201, Lipman Building, Newcastle upon Tyne, NE1 8ST UK; 3 Pacific Action for Climate Transitions, Monash Business School, 900 Dandenong Rd, Caulfield East, 3145 Australia

**Keywords:** Biocultural diversity, Indigenous and local knowledge, Knowledge co-production, Tropical forest fauna

## Abstract

Indigenous and Local Knowledge (ILK) is an expression of biocultural diversity and is vital for inclusive and sustainable forest management and epistemic justice. We examine how researchers studying tropical forest fauna engage with ILK and the Indigenous Peoples and Local Communities (IPLC) who are holders of this knowledge. We conducted a systematic review of 62 articles that focus on tropical forest fauna and ILK. We used a category-based quantitative and qualitative content analysis on the types of forest fauna studied and how research engages with, defines and represents ILK. We also evaluated the varied forms of inclusion of IPLC in the research. We find that less than half of the reviewed studies (25) explicitly define ILK, and only four studies reported including IPLC in the decision-making processes. Our findings reveal that science has not fully acknowledged and understood the depth of ILK and we suggest ways to address this in future research.

**Supplementary Information:**

The online version contains supplementary material available at https://doi.org/10.1007/s13280-026-02378-y.

## Introduction

The role and use of forest fauna in tropical forest ecosystems are increasingly prioritised in research (Krause and Tilker 2022). Tropical forests hold more than half of the known vertebrate species (Pillay et al. 2022) and are crucial for regulating global environmental processes (Khanna et al. 2017; Alves de Oliveira et al. 2021; Boulton et al. 2022; Smith et al. 2023; Flores et al. 2024). Because of their ecological importance, tropical forests have been a focus of ecological, conservation and environmental research, but such research has often obscured that people inhabited and thus shaped these ecosystems for millennia, acquiring profound and sophisticated locally adapted knowledges about these ecosystems and how to use and manage the floral, faunal and other types of material and immaterial resources (Cummings and Read 2016; Marin et al. 2017; Aswani et al. 2018; Paneque-Gálvez et al. 2018; van Vliet et al. 2018; Fonseca-Cepeda et al. 2019; Pironon et al. 2024). These vast knowledge systems and epistemologies can be approached and defined using diverse terminologies. To best capture this diversity, we draw on the broad and inclusive term Indigenous and Local Knowledge (ILK), which was defined by Diaz et al. (2015, p.13) in the Intergovernmental Science-Policy Platform on Biodiversity and Ecosystem Services (IPBES) conceptual framework as “A cumulative body of knowledge, practice and belief, evolving by adaptive processes and handed down through generations by cultural transmission, about the relationship of living beings (including humans) with one another and with their environment. It is also referred to by other terms such as Indigenous, local or traditional knowledge, traditional ecological/environmental knowledge (TEK), farmers’ or fishers’ knowledge, ethnoscience, Indigenous science, folk science”. This inclusive definition highlights the sophisticated knowledge of Indigenous and Local Communities (IPLC) that inform and shape the diverse strategies of IPLC to manage, use and interact with the living and non-living entities in the biosphere.

Given the growing recognition of ILK's importance in biodiversity and conservation research, we carried out a systematic review of how ILK is recognised, conceptualised and defined by researchers working on tropical forest fauna. Moreover, through this review, we want to highlight why it is important for researchers and policymakers to understand that ILK is a crucial biocultural and social–ecological element which is fundamental for epistemic justice and sustainable forest fauna management. Biocultural diversity as a term and framework refers to the variety of life in all its forms: biological, cultural and linguistic, which are interconnected (and possibly coevolved) within a complex socioecological adaptive system that includes the diversity of human cultures and languages (Maffi 2007). This diversity does not exist independently or in isolation; instead, it engages with and influences each other in complex ways. This biocultural dimension of the social-ecological systems (SES) contains diverse knowledge systems, with sophisticated ecological knowledges and management practices historically evolving from independent livelihoods and customary interactions of IPLC with tropical forest fauna (Berkes 2017).

Based on our own research, we approach ILK as a form of knowledge of self-identified individuals and communities, who maintain intergenerational connections to places and nature through their livelihood practices, cultural identity, worldviews, institutions and ecological knowledge (Watson et al. 2019; Krause et al. 2020). We also understand ILK as situated knowledges (Nazarea 1999) and as part of local and informal institutions and norms that shape SES, where people and nature, for instance forests, are intricately linked through direct, indirect and spiritual connections (Berkes et al. 2000; Cajete 2020).

Historically, ILK has been suppressed by states and religious institutions and has been undervalued and underrepresented in scientific studies (Jacobs 2025). As a result of numerous historical reconfigurations, colonisation, persistent colonial practices and rapid social and environmental changes, ILK is increasingly being lost. There is evidence that this loss of ILK follows a general trend that correlates with forest degradation and deforestation, as well as the loss of local languages (Chiblow and Meighan 2022) and the disappearance of entire ethnicities (Aswani et al. 2018; Hua et al. 2019; Cámara-Leret and Bascompte 2021; Bromham et al. 2022). Moreover, the oral intergenerational transmission of ILK has been severely disrupted by broadly expanded colonial structures at the development–education–religion link (Mignolo 2011; Chilisa 2017). Concrete and horrifying examples include religious boarding schools, e.g. Canada, USA (Holiday 2025) and Colombia (Zapata 2008), which led to a severe decline in the relationship between different generations and the transmission of knowledge between elders and young people (Callison et al. 2016). Even though current research is approaching ILK as resilient systems that can also generate new knowledge (Berkes et al. 2000; Gómez-Baggethun and Reyes-García, 2013; Jacobs 2025), it is crucial to recognise that the threats and losses that these systems and their holders have been experiencing for centuries are still ongoing (Tang 2012).

Across the world's tropical forests, people still draw on ILK to use resources and interact with their surroundings. When coupled with associated cultural values, local traditions and spiritual beliefs, these interactions may support the sustainable use of wildlife in tropical forests (van Vliet et al*.* 2015; de Mattos Vieira et al. 2019; Smith et al. 2019; Krause and Tilker 2022). Indeed, the management of tropical forests, particularly viewed through the SES perspective, which recognises an interdependent dynamic between humans and ecosystems, can benefit by incorporating biocultural dynamics by understanding ILK and including its holders in forest fauna management, starting from research practices.

Many scholars have argued that it is essential to recognise and engage with ILK in forest management and conservation efforts (Trosper et al. 2012; Smith et al. 2019; Sunderland and Vasquez 2020; Yoamara et al. 2020; Krause and Tilker 2022). The crucial role of IPLC and their knowledges has also been emphasised by the IPBES and IPCC (Ford et al. 2016; McElwee et al. 2020), which underlines the need for decolonial research approaches where these knowledges are not only recognised and receive more attention, but where the knowledge holders are protagonists in co-production and in the validation of the representations of their knowledge (Whyte 2013; Schröter et al. 2023; Jacobs 2025; Subramanian et al. 2025). Nonetheless, this is only possible if knowledge co-production and epistemic justice are assured and prioritised (Meisch 2024; Jacobs 2025).

Other key co-production proposals use “weaving of knowledge” as a term proposed in Tengö et al. (2017), which refers to co-production processes based on respectfully recognising and bringing together both ILK and Western knowledges in collaborations that respect the integrity of each knowledge system towards societal challenges. Increasingly, sustainability scientists have argued that solving current social–ecological crises requires an iterative process of societal change that values and centres different ways of knowing, being and acting (Wyborn et al. 2019). Sustainability practitioners are also increasingly recognising fundamental principles of co-production in research and management, which are defined by Nostrom et al. (2020, p.183) “as an iterative and collaborative process involving diverse types of expertise, knowledge, and actors to produce context-specific knowledge and pathways towards a sustainable future”.

Social and environmental justice underpins sustainability science (Ott and Döring 2011; Grunwald 2020; Martínez-Alier 2023). Thus, there is a need to recognise and address the systematic oppression and exclusion that ILK has suffered over centuries, which have lasting consequences for sustainability, transdisciplinary research practices and the ability to address epistemic injustices that continue to this day (Mignolo 2011; Chilisa 2017). Therefore, transdisciplinary and transformative sustainability research must ensure that knowledge co-production includes relevant knowledge holders and meets the aspiration of epistemic justice (Meisch 2024). With regard to our own research, the ILK of tropical fauna must inherently be part of knowledge co-production processes for its sustainable use and management in tropical forests. ILK of forest fauna is the overarching approach that we used in this review as its inclusion in research has implications for the management of forest fauna—both from within an ILK perspective and local resource users (hunters, fisher communities, etc.) and for a conservation managers or national authorities who seek to align objectives of ecosystem protection and sustainable resources management with local needs and forms of use.

While we are not Indigenous scholars and do not claim any insider knowledge of ILK, we seek to answer the following research question: How are researchers studying tropical forest fauna and management engaging with IPLC and their respective ILK? We answer this question through our systematic review by first outlining (1) geographical distributions and frequencies of studies, including forest fauna types, and then considering (2) how ILK on tropical forest fauna is defined and approached in the studies and (3) to what extent research on ILK is based on collaboration and co-production with the knowledge holders and IPLC in places where the research is carried out. By doing so, we provide a state-of-the-art approach in the field and provide evidence, gaps and omissions that scientists and practitioners can use to advance a research agenda grounded in and encouraging deeper engagement with the ILK of IPLC in research on tropical forest fauna.

## Materials and methods

### Defining the review, terms and filters

**Fig. 1:**
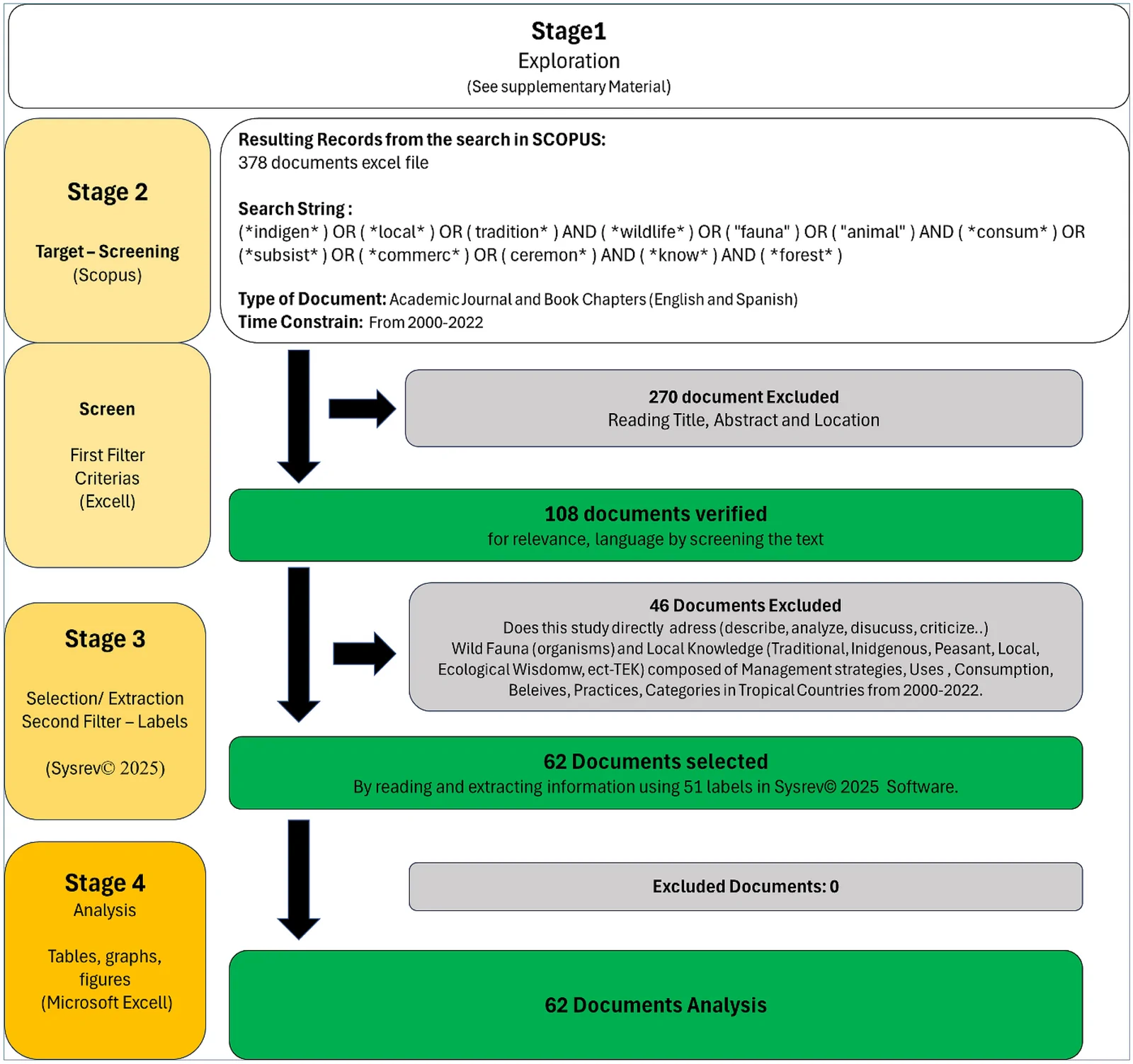
Overview of Forest Fauna ILK Systematic Review Process

We conducted a systematic literature review and used a four-stage process to identify, obtain, save and process data (Fig. 1): (1) we carried out an exploratory search in EBSCOhost and Scopus to get an overview of the extent of research on the topic and developed a comprehensive search string; (2) we designed a specific and targeted screening process to identify relevant articles in Scopus; (3) the relevant articles were selected and extracted using Sysrev © 2025 Insilica LLC Software; and (4) in a final step, we read the selected articles in Sysrev and extracted data according to pre-defined categories related to the research objectives and then analysed the data in Microsoft Excel.

After testing various search strings, which refer to different combinations of search terms, we selected a search string that yielded a sufficiently extensive and relevant list of forest fauna literature from the past twenty years. After the Exploration Stage 1 (see Supplementary material S1), the Search String Selection and Screening (Step 2) used the following combination in Scopus search engine looking for titles, abstract and keywords containing the following terms: (*indigen*) OR (*local*) OR (*tradition*) AND (*wildlife*) OR ("fauna") OR ("animal") AND (*consum*) OR (*subsist*) OR (*commerc*) OR (*ceremon*) AND (*know*) AND (*forest*).

This yielded an initial set of 378 articles published between 2000 and 2022, which we then screened for relevance and duplicates, resulting in 108 articles after the second filter. We applied further exclusion criteria to filter out documents with incomplete or insufficient data, or where the content, topic or discipline fell outside our scope (e.g. traditional trade—commerce, indigenous species—phylogenetics and tropical and indigenous parasites—medical taxonomy, marine and fish industry).

We uploaded the final list of 108 documents to the Sysrev© 2025 platform and reduced the selection to 62 publications, published between 2000 and 2022, ensuring their relevance as they: (1) occurred in tropical regions; (2) addressed local knowledge of fauna; and (3) mentioned IPLC and ILK (Fig. 1).

### Scope, inclusion and exclusion criteria

The screening process and selection of information were done using Sysrev © 2025 Insilica LLC Software, through a list of 51 labels grouped into seven categories to select and compile qualitative and quantitative information (Supplementary material S2). We obtained data according to the following predetermined categories and labels: (1) explicit definition and description of ILK, (2) authors' literal description in the document of the objective and methods that explain the degree of community participation and (3) description of diversity of fauna and the types of use given by the community. Publication information, such as author, funding, location and ecosystem categories, was also included. Once all the filtered publications were read and the respective information obtained, we transferred the data to Microsoft Excel for further quantitative analysis, including distribution, frequency, trends and visual representation. The qualitative categorisation of the degree of participation was conducted by searching for, reading and quoting the author's direct descriptions of IPLC involvement, and the results were presented in comparative tables.

## Results

### Distribution of publications in time

**Fig. 2:**
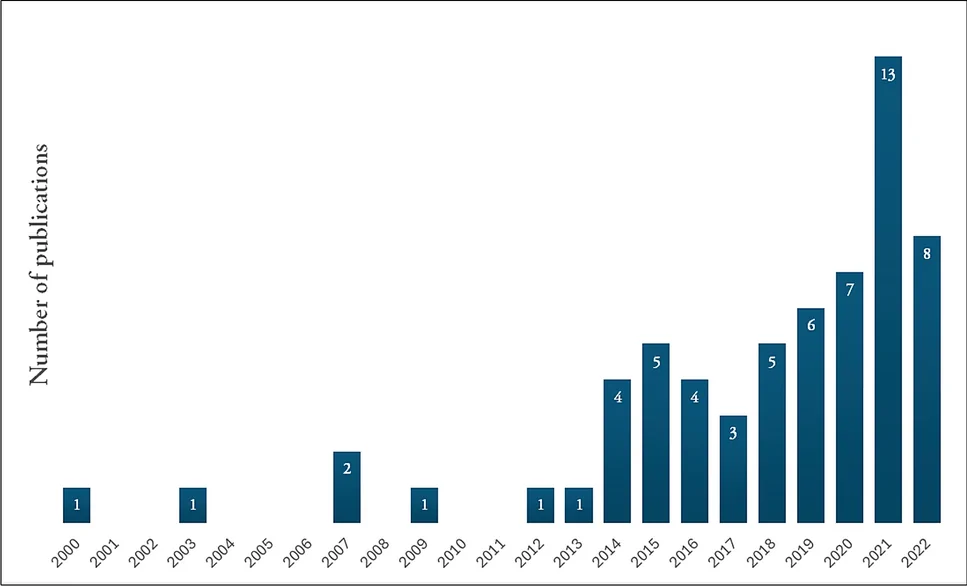
Number of published articles studying tropical forest fauna, ILK within IPLC in peer-reviewed journals (62 articles between years 2000 and 2022)

We found a clear trend of a gradually increasing number of publications about ILK on forest fauna over the last 22 years, with seven publications (11.2%) published between 2000 and 2013 and the majority from 2014 onwards (88.7%) (see Fig. 2). Although ILK gained some prominence in the wider environmental studies literature after 2000 (Berkes et al. 2000), there appears to be a delay before researchers working on tropical forest fauna began to engage with the concept. The rise of publications post-2014 demonstrates a consistent and growing interest in ILK. Although we cannot be certain, the marked increase in publications from 2014 may correspond to the work and attention of the IPBES, which has recognised and highlighted the importance of ILK of IPLC to manage and protect biodiversity sustainably.

### Studied countries

**Fig. 3:**
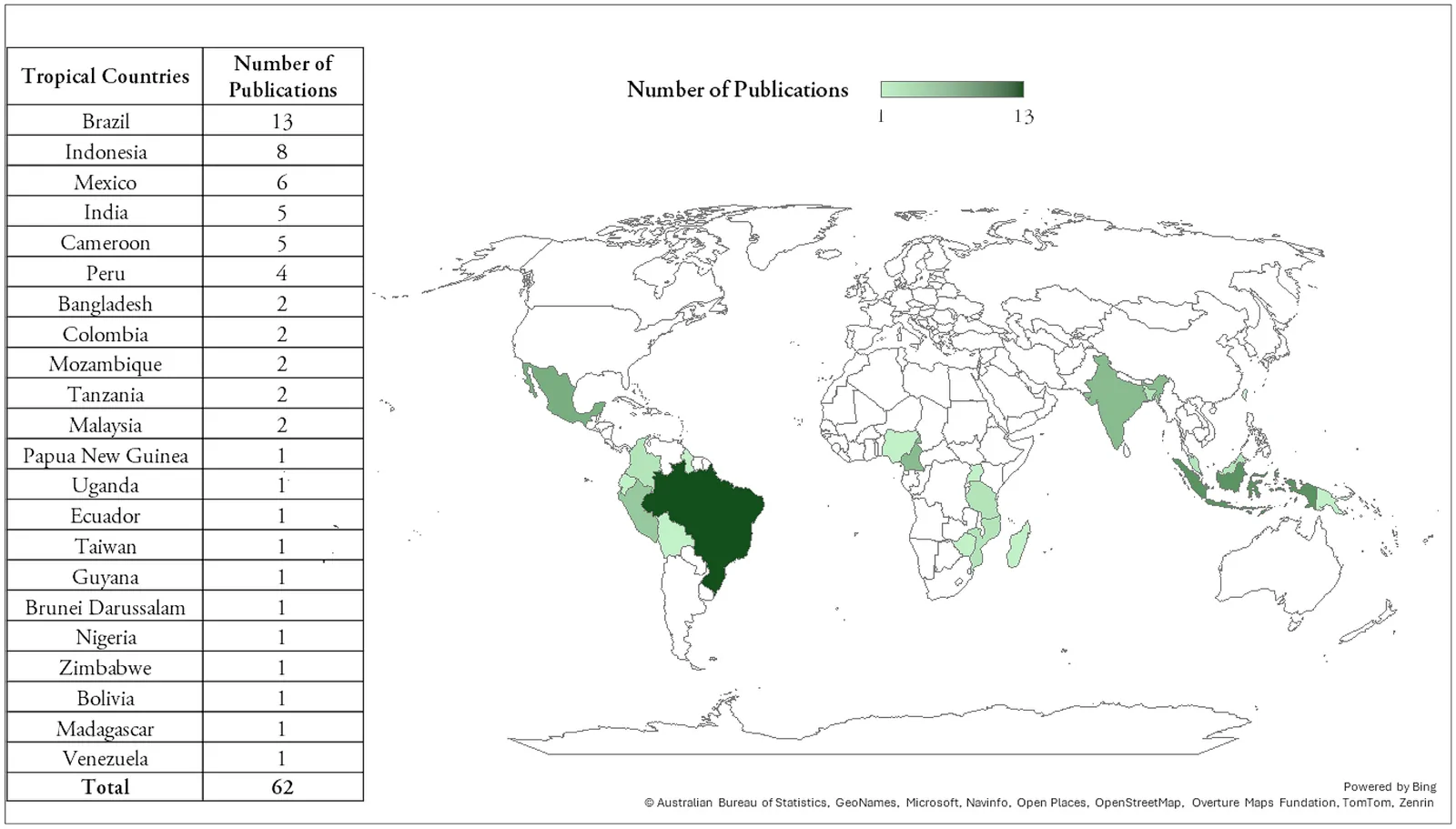
Number of publications in tropical countries and their global distribution

The analysed studies were conducted in 22 tropical countries with an uneven distribution. Brazil was the most represented country, with 13 studies (20%), followed by Indonesia (*n* = 8, 13%), Mexico (*n* = 6, 9.8%), India (*n* = 5, 8%) and Cameroon (*n* = 5, 8%) (Fig. 3). The other 17 countries are represented by one or two publications each, with an evident absence of research occurring in Central America (except Mexico) and sub-Saharan Africa. In South and Southeast Asia, Indonesia (8) and India (5) were the most studied countries. This uneven geographical distribution highlights the need to expand research to understudied geographical ranges to ensure the representation of ILK of the thousands of different IPLC that live across the tropical forest realm. However, while we did not find publications from countries where one would expect this topic to be highly relevant, such as the Democratic Republic of Congo, Venezuela, Lao PDR or Myanmar, this does not mean that such studies do not exist, but they may be written in another language or published in journals not listed in Scopus. Another explanation may be the difficulties researchers encounter when conducting this type of research in certain countries. Brazil is the most represented country, likely due to its size, relatively well-established research infrastructure and the long-standing history of research on TEK and forest fauna in the Amazon Basin.

### Fauna types and species

**Fig. 4:**
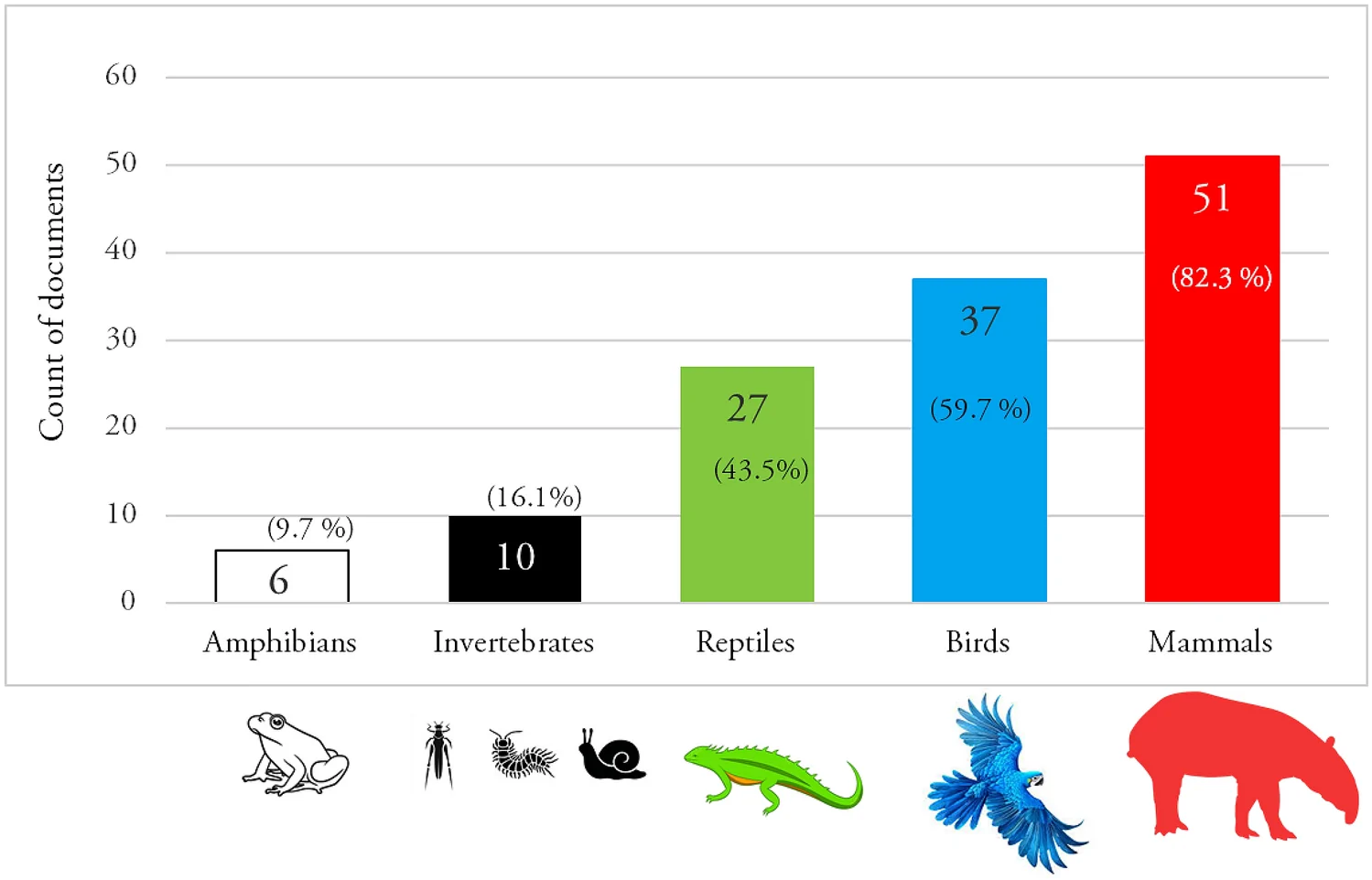
Number of documents and percentage (%) mentioning each different classes of fauna

The different species of forest fauna that were studied in the selected publications are described using common (vernacular) and scientific names (binomial) (in two cases using local language), and grouped into five categories: Mammals species are named in 51 documents (82.3%), followed by birds (*n* = 37, 59.7%), reptiles (*n* = 27, 43.5%), invertebrates (*n* = 10, 16.1%) and amphibians (*n* = 6, 9.7%) (Fig. 4).

**Fig. 5:**
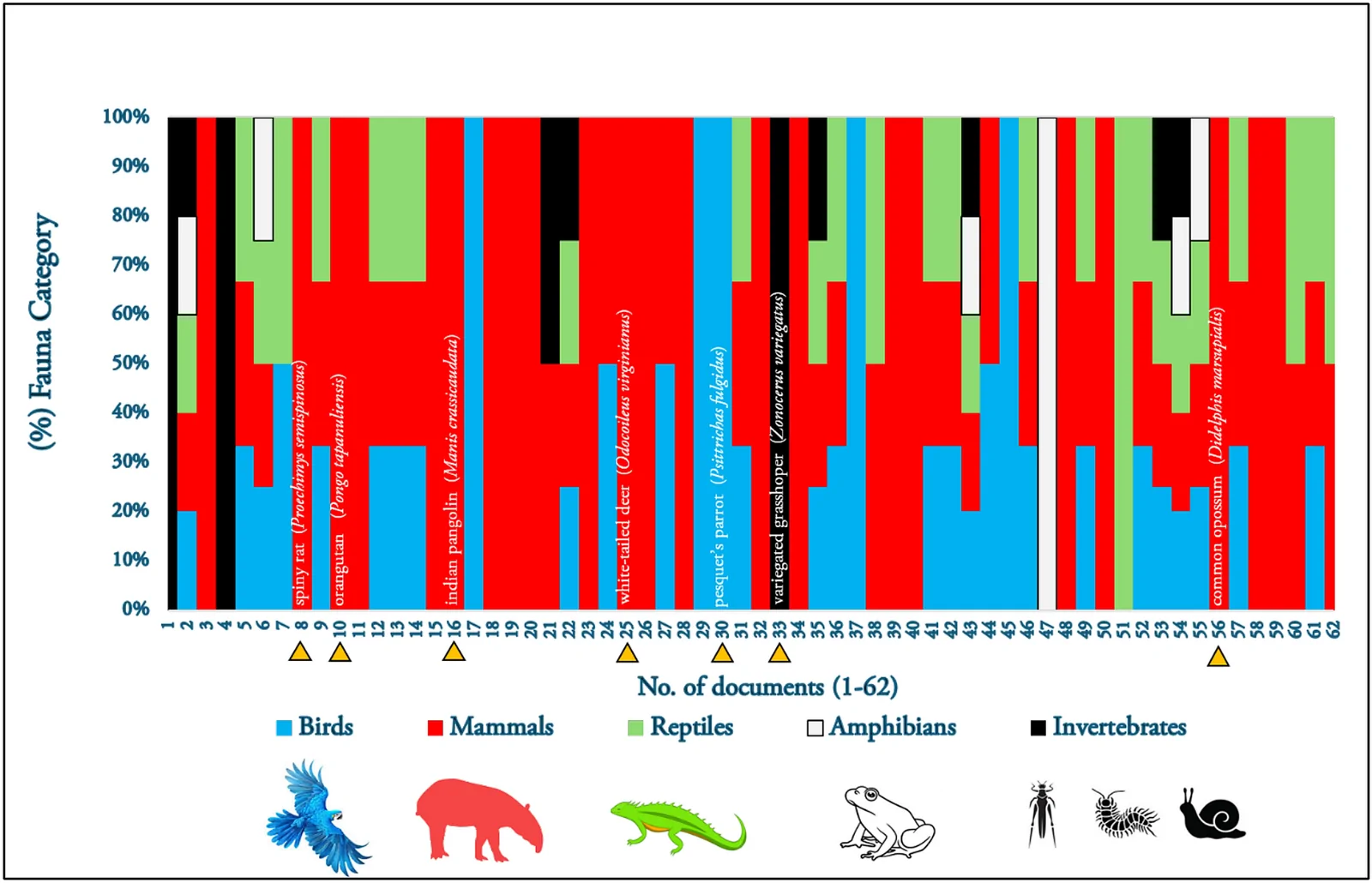
Forest Fauna types' percentage composition for each study. Studies describing a single species are marked with a triangle and both vernacular and (*binomial*) names

To understand how the differences in Fig. 4 are represented within each document and compared to the others, we present the fauna composition for each of the 62 publications in Fig. 5, listed in order on the x-axis (numbers 1–62). The figure illustrates variations in the type of fauna analysed, including studies focusing solely on one species.

While 32 documents describe more than one (> 1) fauna class, 30 publications describe only one class of fauna. Twenty-three publications exclusively focus on mammals, five documents exclusively focus on birds, three exclusively focus on invertebrates and one exclusively focuses on reptiles and amphibians. From the 62 documents, 55 referred to more than one species and 7 studies were exclusively describing a single species (monospecific publication—yellow triangle—see Fig. 5), such as document No. 10 on the Tapanuli orangutan (*Pongo tapanuliensis*) or document No. 16 on the Indian pangolin (*Manis crassicaudata*) (Fig. 5).

### Types of uses of forest fauna

We analysed the selected publications regarding the different types of uses of forest fauna that were reported. Nine types of explicit use of forest fauna were registered in the 62 publications, ranging in frequencies from ¨Edible¨, as the most common reported use of tropical fauna (*n* = 56, 90.3%), followed by commerce (*n* = 29, 46.8%), medicine (*n* = 29, 46.8%), ceremonial (*n* = 27, 43.5%) and ornamental (*n* = 12, 19.4%) categories (see Table 1).

**Table 1:**
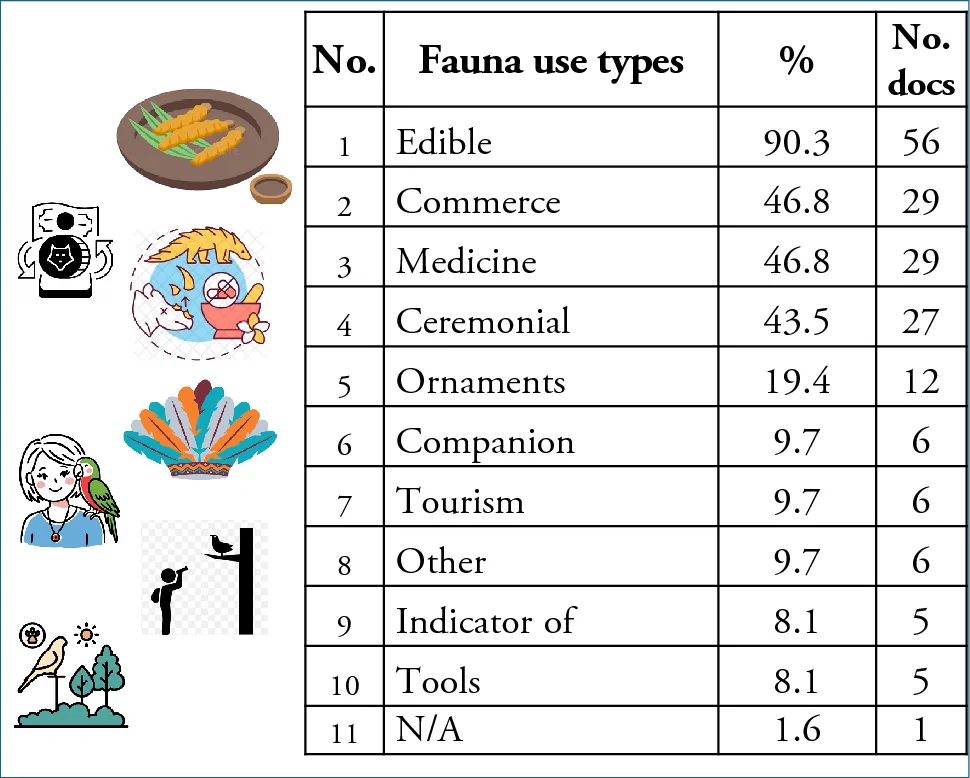
Type of Uses reported for Tropical Fauna in 62 documents

These “fauna use” categories were also reported in different combinations (42 combinations in 62 documents) within the included studies. “Edible” is present in 56 publications and is often combined with other uses in 82% of the studies. Exclusive edible use is reported in 13 publications, followed by edible use in combination with commercial, medicinal or ceremonial uses (*n* = 6, 4, 2, respectively). The remaining combinations are reported only once (see Supplementary material S3). Table 2 illustrates examples with direct quotes from articles that describe different types of management and use of forest fauna.

**Table 2:** Details of the types of use and management of Forest Fauna

**Type of Use** Activity (Purpose)	**Extracts of use and management of fauna as described in the articles**	**Country** *Document Number in* *S6**list*
**Edible Fauna** Subsistence Hunting (Food and Crop Management)	“Because of their strong dependence on hunted wildlife for protein, the Mro have an intimate understanding of the relationships between these animals and their habitat”. p.61 The survey revealed that the Mro hunt for two purposes: for highly valued game meats and to protect their crops in the Jhum land from wild animals and birds” p.59	**Bangladesh** (Chowdhury et al. 2007) *No.2*
**Edible Fauna** Insect Harvest (Nutrition Well-being)	“We observed that Guajibo living at the savannah border (at Alcabala Guajibo, Amazonas, Venezuela) relied mostly on insects (especially grasshoppers and larvae of Rhynchophorus palmarum) during the rainy season of July to August and estimated that over 60% of their animal protein came from insects”. p.2251 “The Ye’Kuana consumption of earthworms cannot be attributed to a lack of other animal protein resources because there is no shortage of fishes or game in their territory (Schlenkner 1974; Hames 1980). Rather, the Ye’Kuana appreciate these species of earthworms as gourmet foods”. p.2249	**Venezuela** (Paoletti et al. 2000) *No.4*
**Edible** **Medicine** **Tools** **Ornament** **Ceremonial** Hunting (Food, Well-being)	“Thirty-nine species that provided raw materials (fat, bones, feathers, viscera, body fluids) for the production of zootherapeutics were mentioned, notably the fat of S. merianae”. p.258	**Brazil** (Barbosa et al. 2022) *No.6*
**Edible** **Commerce** Hunting, Weddings, Funerals (Food, Well-being)	“They [two local communities of the Ruvuma landscapes] hunt small mammals for subsistence purposes (meat) or bigger animals (for example elephants) for income generation or traditional events (such as providing meat in weddings or funerals for a large number of people)”. p.858	**Mozambique, Tanzania** (Zafra-Calvo and Moreno-Peñaranda 2018) *No.42*
**Medicine** **Signal of** **Ceremonial** Capture, Observation, Interpretation (Well-being, prepared by Socioecological Predictions)	“The category of climatic and environmental changes predictors (ECP nine species) is understood as the reading and interpretation of a signal expressed from an unusual song or activity of the bird, which indicates a change in the weather or a specific climatic event” p.8 “Environmental Signal, Rituals, Omes, Medicine: e.g. help them to interpret rain announcements that allow them to prepare actions; for instance, the “salineros” rush the collection and protection of salt in the face of this rain forecast “. p.9	**Mexico** (Romero-Bautista et al. 2020) *No.29*

Apart from these explicit uses, other variations and inferred uses were also reported. Some of these fauna values were either placed in the “Other” category or left unanalysed (see, for instance, “pest/spirit animal” (Harahap and Ridwan 2022) and “trophy hunting” (Zafra-Calvo and Moreno-Peñaranda 2018). One example of these nuances is the intrinsic value of bats as an aesthetic experience, such as the “pleasure of seeing bats flying”, apart from their edible, medicinal and ornamental tourism uses, as reported by Rocha et al. (2021). Although forest fauna was identified as being used primarily as a food resource, the variety of multiple material and spiritual uses exemplifies the intricate relationship between humans and animals in tropical forest social–ecological systems. The use of fauna as medicine and in ceremonies shows its spiritual importance for people who rely on their ILK to manufacture these medicines and to perform diverse and sophisticated ceremonies that sustain their communities’ physical and psychological well-being.

### Indigenous and local knowledge definitions and author’s positionality

As previously noted by other authors, ILK lacks a unified, consistent definition or acronym, as reflected in our review (Whyte 2013; Berkes 2017; Khanna et al. 2017; Jacobs 2025). The 25 documents that made direct reference to ILK reveal a diversity of terminology when referring to ILK, with 10 distinct acronyms (43% of the total studies). Our understanding of ILK recognises this diversity and includes other terminologies such as Traditional Ecological Knowledge (TEK), Local Environmental Knowledge (LEnv.K) or Ecological Wisdom as part of ILK (see Supplementary material S4). More interestingly though, the remaining 37 studies provided no explicit definition of ILK or mention of other types of IPLC knowledge.

We observed a tendency that studies that did not explicitly define ILK were more likely to be less participatory and inclusive of IPLC. These studies primarily focused on scientific methods to analyse population dynamics of species of tropical forest fauna (Hallett et al. 2019) or measure the potential and actual impact of road development on these populations (Pattiselanno and Krockenberger 2021), but did not mention active collaboration with IPLC nor engagement with ILK in their analyses.

Aligned with our objective to examine how ILK on tropical forest fauna is characterised and approached by academic publications, we searched for the author's position on ILK expressed directly or inferred from the selected publications. We present examples of the terms used or cited by authors, along with their positions and descriptions of ILK. We include details of the respective country of study, the specific references used in the document and the document number (see S6 for the complete list) in Table 3.

**Table 3:** ILK definitions and the author's approach to ILK

**Knowledge Terminology Used**	**Direct quotes of the authors' approach to ILK**	**Country** (Reference) Number of the reviewed document in list S6
**Local Ecological Knowledge (LEK)** “… can be defined as all the personal empirical experience that local people acquired through years of interactions with natural environments, which are transmitted to their communities” (Charnley et al. 2007; Brook and McLachlan 2008)	“Community-based management of natural resources has been considered the best available option for the local protection and/or recover [sic] of native biodiversity, in addition to socioeconomic gains in enhancing the living standards of rural populations” p.9	**Brazil** (Sampaio et al. 2022) *No.7*
**Local Environmental Knowledge (LEnvK)** “LEnvK linked to birds is transmitted generationally, from grandparents and parents to children and grandchildren” (Romero-Bautista et al. 2020)	LEnvK is needed: “To understand the various human-bird environmental interactions, it is necessary not only to address the utilitarian importance that species have in a specific place but also to address the importance of intangible cultural relationships and read the connection between these aspects, by resorting to it to the uses and to the system of beliefs and normativity that persist and resist” p.12 “This teaching–learning process is linked to experience and observation of the environment; it is transmitted while walking and working the field, through daily observation and the assessment, use and conservation of species” p.11	**Mexico** (Romero-Bautista et al. 2020) *No.29*
**Local Traditional Knowledge (LTK)/Local Indigenous Knowledge (LIK)** “most LTK themes focused on species-specific knowledge rather than the social and cultural contexts of campesino hunting” (Petriello and Stronza 2020)	“Rather, we recommend greater consideration of other marginalised and disenfranchised groups and their relevance to conservation” p.351	**Mexico, Peru, Colombia** (Petriello and Stronza 2020) *No.31*
**Not Specific terminology (NST)** mentions but does not describe LK “Local calendars were also related to religious and natural markers, acting as parameters for the livelihood activities” (Hanazaki et al. 2009)	“As stressed by (Redford 1991), native inhabitants should not be considered as ecologically noble savages, despite their deep knowledge about the environment. p.7 “The great challenge now is finding ways to cope [sic] the recent changes affecting these local people and changing their livelihoods and their values, with factors such as the maintenance of the social conditions of production of this local ecological knowledge and the improvement of their life quality”. p.7 “Local Indigenous knowledge on native resources is important to promote local development in a sustainable way (Gadgil, Berkes et al. 1993), and help to conserve biodiversity, particularly if the resource is used”. p.1	**Brazil** (Hanazaki et al. 2009) *No.61*
**Not Specific terminology (NST)** mentions but does not describe LK	“Conservation and public health interventions in such settings may be most efficient when they capitalize on local knowledge and target root socio-economic and cultural drivers that lead to hunting behaviour”. p.1	**Nigeria** (Friant et al. 2015) *No.62*
**No specific terminology (NST)** “Detailed knowledge”	“The Indigenous people's strategy in the consumption of invertebrates indicates that they have a detailed knowledge of their environment, in the use and manipulation of forest resources to provide food for human populations, without the destruction of biodiversity, a practice that has been maintained through millennia” p.322 “Knowledge of the relationships between Indigenous populations and the ecosystem represents the basis for a natural and cultural preservation of biodiversity”, p.322	**Venezuela** (Araújo and Beserra 2007) *No.1*
**Traditional Ecological Knowledge (TEK)** “Is a cumulative body of knowledge, practices, and beliefs that has evolved by adaptive processes and has been handed down through generations by cultural transmission” “It is about the relationship of living beings (including humans) with one another and with their environment” (Berkes 1993)	“To successfully incorporate TEK into natural resource management, authorities should be concerned about the gradual loss of TEK, and we suggest that authorities should actively increase the investigation of TEK and documentation through written records, which is vital before it is lost forever”. p.218 “Application of TEK to natural resources co-management with aborigines has recently become a policy trend. It is therefore necessary to know how TEK can contribute to natural resource management”. p.214	**Taiwan** (Wu et al. 2014) *No.57*

The opening lines of Agnihotri et al. (2021, p.1), stating “The traditional ecological knowledge (TEK) of Indigenous communities is nowadays regarded as a valuable resource in efforts to conserve biodiversity”, capture the predominant relation many authors hold regarding ILK. Whereas ILK was widely considered a valuable tool for science, governments and NGOs in their efforts to halt biodiversity decline in tropical forests (e.g. Friant et al. 2015; Petriello and Stronza 2020; Piña-Covarrubias, 2022), little attention is given to ILK as an intrinsic component and expression of the biocultural diversity of tropical forests seen from a social–ecological systems perspective.

Piña-Covarrubias et al. (2022), for example, recognise that TEK could be used for species conservation or sustainable development. In addition, Pangau-Adam et al. (2022) also recognise that traditional land conservation practices based on ILK are a valuable resource for managing introduced rusa deer populations in West Papua. These examples reflect the standard representation of ILK in the selected publications that recognised and named ILK or related terms. Although the recognition of ILK's value for research is a positive first step, such research also risks instrumentalising ILK and reducing its value to merely serving externally defined goals, such as Western ideas of conservation and sustainable development.

Only a few studies fully recognised ILK as more than just another data point. For instance, authors like Romero-Bautista et al. (2020) provided more profound insights and clear evidence of the value of such research, recognising the immaterial value of ILK and demonstrating a sensitive and keen awareness of the multidimensionality of fauna–human relations:

“To understand the various human-bird environmental interactions, it is necessary not only to address the utilitarian importance that species have in a specific place but also to address the importance of intangible cultural relationships and read the connection between these aspects, by resorting to it to the uses and to the system of beliefs and normativity that persist and resist”. (Romero-Bautista et al. 2020, p.12)

Thus, beyond the recognition of ILK as a scientific and expression of biocultural diversity of IPLC, it is part of the local governance and management system that has enabled the sustainability of the use of fauna for medicine, food and tourism.

### Inclusion of indigenous people and local communities in research

**Fig. 6:**
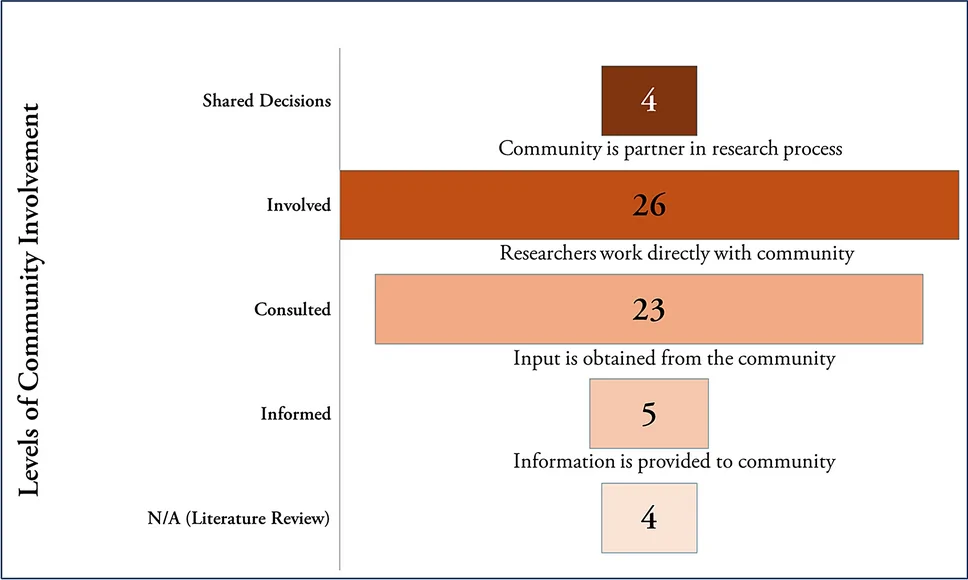
Distribution of studies (n = 62) in our five categories of Community Involvement in ILK fauna studies

To answer our research questions, we have considered the degree of local or community participation as a variable that reflects the current ways in which academia approaches and interacts with ILK (Vaughn and Jacquez 2020). For this reason, we analysed the selected documents using five criteria of community participation adapted from the International Association of Public Participation (IAP2) spectrum of public participation (IAP2 2018) and Vaughn and Jacquez (2020). We scrutinised respective methodologies and categorised the documents according to the degree of local community participation. Figure 6 shows the level of inclusion of IPLC across our sample, and we provide examples of the distinct levels of participation we identified in the included studies (Supplementary material  S5).

Agnihotri et al. (2021) and Akumsi et al. (2003), for instance, describe how IPLC were part of shared decision-making as follows:

“This paper is not intended as an objective, empirical investigation of Solega people’s attitudes toward, and practices regarding, conflict with wild animals. Instead, we present the following information with a two-fold aim. The first is to give a voice to Solega people, as Indigenous voices and points of view are often absent from discourses surrounding endangered wildlife, invasive species, and general biodiversity conservation” (Agnihotri et al. 2021, p.4).

“The development of the strategy emphasized the involvement of hunters and other traditional authorities, and the process involved adapting and testing existing local wildlife management systems” (Akumsi 2003, p.38).

In contrast, the study by Pangau-Adam et al. (2022), which we categorised as “Consulted” IPLC, states:

“Here, our aim was to assess the population status of rusa deer, and to investigate the extent of hunting practices on this mammal in West Papua...We conducted camera trapping and line transect surveys simultaneously to estimate...We also interviewed hunters (n = 134), informants (n=9) and households (n=91) to assess hunting patterns and socioeconomic importance of rusa deer across 15 districts of the Tambrauw regency”. (Pangau‐Adam *et al.*, 2022, p.1).

A representation of the distribution according to the degree of participation is presented in Fig. 6.

Many studies directly involved (*n* = 26) or consulted (*n* = 23) IPLC, but only four reached a level of inclusive and participatory scientific research where IPLC were able to influence decisions on the scope, content and methodology of the research. This distribution is, unfortunately, not surprising and repeats a long-standing pattern in ecological and biological field research, where scientists rely on IPLC logistical support to identify and access ecosystems for studying biodiversity. For this reason, we identified only four studies that explicitly employed a participatory action research approach, in which the research was co-designed and subsequently carried out in close collaboration with community members who retained ownership of the knowledge (Table 4).

**Table 4:** Details of sharing a decision-making approach in articles (co-production)

**Article Author and Year** **Title** (Number of the reviewed document in list S6)	**Details of Shared Decision Studies** (Communities are partners of study)
**(**Akumsi 2003**)** Community participation in wildlife management: the Mount Cameroon experience. *Unasylva*, 54(3), 37–42 (No. 3)	“The objectives of the strategy were: to create village-based institutions and strengthen their capacity for managing wildlife sustainably to develop locally defined rules and regulations that can support sustainable management efforts based on local realities and within the confines of the national wildlife legislation to define community hunting areas and sustainable offtake to develop a simple local monitoring and evaluation system with the participation of all stakeholders” p. 38
**(**Zafra-Calvo and Moreno-Peñaranda 2018**)** Exploring local people's views on the livelihood impacts of privately versus community managed conservation strategies in the Ruvuma landscape of North Mozambique–South Tanzania. *Journal of Environmental Management*, 206, 853–862 (No. 42)	“The aim of this study is to identify a strategy that achieves sufficient levels of nature protection while being perceived as acceptable by the communities in terms of livelihoods costs and benefits, and thus a strategy that communities would be willing to commit to” p. 854
(Franco and Minggu 2019) When the seeds sprout, the hornbills hatch: understanding the traditional ecological knowledge of the Ibans of Brunei Darussalam on hornbills*. Journal of ethnobiology and ethnomedicine*, 15, 1–14 (No. 37)	“Hence, we conceived this study to understand the TEK of the Iban community of Temburong in Brunei Darussalam on their hornbills and the cultural importance accorded to them” p. 2
**(**Agnihotri et al. 2021**)** Tiger becomes termite hill: Soliga/Solega perceptions of wildlife interactions and ecological change. *Frontiers in Conservation Science*, *2*, 691,900 (No. 22)	“This paper presents aspects of the TEK of the Soliga/Solega1 community of southern India, who live in a forest habitat that is also home to tigers, leopards, elephants and sloth bears, among others” p. 2 ***This case was visited by one of the authors by coincidence, as the lead author was invited to the Soliga community by an ATREE college in January 2025, confirming the characteristics that make this process a collaboration between academia (ATREE) and Soliga Indigenous community

## Discussion

In our review of 62 publications (see complete list in Supplementary material S6), we revealed how researchers studying tropical forest fauna engage with IPLC and their respective knowledges. While this topic has been receiving increasing attention over the past two decades, we noted a spike since 2014, coinciding with the release of the IPBES reports, which highlights the role and importance of ILK for biodiversity conservation (Díaz et al. 2015; Tengö et al. 2017; Pörtner et al. 2021). Nonetheless, the majority of studies focus on the instrumental value of ILK and not so much its intrinsic value, nor the significance and interconnectedness with biocultural diversity. While few studies are explicit about how the authors understand or engage with ILK in their research, our findings indicate that there is considerable room for improvement in research studying the role and use of forest fauna in tropical forest ecosystems, even when ILK and its holders are claimed to be part of the research and management decisions.

In the fauna dimension, an interesting finding of our review is the focus of studies on mammalian and avian species, while most of tropical forest faunal biodiversity comprises invertebrates. This human lens and interest in species with hair and feathers have been observed in previous studies (Morris et al. 1995; Serpell 2004; Batt 2009; Byrd et al. 2017), but they may also portray a skewed picture of ILK in relation to tropical forest fauna as the mammalian and bird bias is likely enhanced by the non-inclusion of ILK. Based on our own experience and previous research (Rosso-Londoño, 2013; Yoamara et al. 2020), we are aware that a vast wealth of ILK on invertebrate species and amphibians exist, yet it is rarely reflected in the published literature.

We understand the uneven distribution of research in countries originates from complex dynamics unrelated to exclusively biological factors. If academic research were a direct match for biocultural diversity (a possible proxy for ILK occurrence), Cameroon, Colombia and Papua New Guinea would be competing for first place on the list (Maffi 2007). However, these highly bioculturally diverse countries (except for Brazil, which is the most studied country in our sample of 62 articles) appear only after the 4th position in the list (Cameroon, five articles; Colombia, three articles; and PNG, one article). A possible explanation for this can be the direct colonial relation of university structures and dynamics to its European origins, which contribute to unequal approaches to knowledge that replicate the subjugation of ILK in tropical territories and colonies (Carvalho and Flórez-Flórez 2014). Other factors, such as the country's position towards rural livelihoods, ethnic diversity and IPLC, can also be reflected in the interest and willingness to research ILK. Again, even though Brazil shares a shameful story of massive destruction of its ecosystems and cultures, it has also been an example of “grassroots” social movements to protect Indigenous territories and the development and divulgation of ILK, which is demonstrated by the influential work of Ailton Krenak, Sônia Guajajara, Eduardo Batalha Viveiros de Castro and Davi Kopenawa. In many highly biodiverse countries throughout the tropics, violent conflict is also an important barrier to carrying out continued research on and with ILK and forest fauna. The past decades and current security situations in specific regions of Colombia or Cameroon are examples of how armed conflicts erodes the possibility of long-term research in regions with a high biocultural diversity (Weir et al. 2024; Garcês and Pires 2025).

Regarding ILK definitions, we highlight two tendencies in our review. The first shows diverse definitions of ILK, with TEK and combinations of Local Traditional Knowledge (LTK)/Local Indigenous Knowledge (LIK) being the most used. The second tendency relates to the non-definition or non-formal naming of ILK, as demonstrated by only 25 studies out of 62 that define it. When defined, however, we observed some interesting and uncommon terminologies, including Ancestral Knowledge, Local Wisdom and Strategic Relational Symbiosis. Each of these definitions attempted to highlight specific representations of ILK in the respective contexts. In our view, such definitions help illuminate the diverse understandings of the world, connecting knowledge with ancestry and fostering awareness of the interdependence of human life with other creatures. This diversity and focus on relational and transcendental descriptions of ILK are linked to how Indigenous scholars defined and described ILK, with a focus on relationship, responsibility and reciprocity to manage fauna (Jacobs 2025).

Lastly, we also found that the level and extent of inclusion and participation of IPLC in research on tropical forest fauna appear largely consultative, symbolic or superficial, rather than fully collaborative and co-produced. There may be several reasons for this, such as a lack of awareness, resources and time to engage in transdisciplinary research and knowledge co-production. Moreover, conducting co-production research requires interdisciplinary teams of researchers who can navigate the complex ethical, social, cultural and ecological aspects that this deeply collaborative research entails, as well as dedicating the time, energy and financial resources required for such a process (Viswanathan et al. 2004; Koster et al. 2012; Gittelsohn et al. 2020). The limitations set by traditional scientific research and its institutions are likely a major hurdle. However, we were nonetheless surprised that few papers even acknowledged these limitations and outlined a path for potentially more inclusive and participatory research.

We found that research still lacks a co-production approach with IPLC and exemplify this with our review of studies on ILK and forest fauna. Knowledge co-production with IPLC is crucial for several reasons. Firstly, ILK can offer significant explanatory contributions to Western science (Brodnig and Mayer‐Schönberger 2000; Maffi and Woodley 2012; Castello et al. 2024), which recognises the limitations of any particular knowledge systems in understanding and holistically addressing the complexity of any given phenomenon. This is the case for the use and management of tropical forest fauna that affects the functional integrity of tropical forests and their role in the global carbon cycle (Galetti et al. 2017; Berzaghi et al. 2018; Gardner et al. 2019; Fricke et al. 2022). Second, epistemological and decolonial arguments highlight the ethical and justice dimensions of knowledge co-production in research that must bring benefits to IPLC in addition to value for external institutions (Edwards and Heinrich 2006; Chilisa 2017). Such arguments are underpinned by sociopolitical ambitions and the rights of IPLC, such as knowledge recognition, identity, sovereignty and self-determination (Agrawal 1995; Aikenhead and Ogawa 2007; Bennett et al. 2025) and claims that research can and should contribute towards achieving these goals. Third, and finally, there is a practical need for ILK for the efficacy of promoting transformative actions in the knowledge-to-action boundaries, where practices and policies need to engage and be co-produced with ILK to be able to produce salient, credible and legitimate information and solutions (Cash et al. 2003; Matuk et al. 2020; Pörtner et al. 2021). Considering our findings, these arguments on effective and transformative actions for sustainability must integrate explanatory, epistemic and legitimacy dimensions of ILK.

Legitimacy of data, and hence results, is crucial to produce information and technology. To be legitimate, knowledge co-production ought to be respectful of stakeholders’ divergent values and beliefs, be unbiased, and fair in its treatment of opposing views (Cash et al. 2003). Legitimacy occurs when all participants (e.g. IPLC, academics or policy practitioners) consider the outcomes of knowledge co-production to be valid according to the diverse meanings and contents of their knowledge systems (Lam et al. 2020). This validity in relation to effective and legitimate actions brings into clear view the critical importance of profound dialogues with ILK and the translation of concepts, views, causes and effects of the sustainability challenge addressed. This is clearly supported by our review results, which show that studies on forest fauna and ILK include wide topics ranging from food consumption, forest governance, to road development and tourism, all of which require knowledge co-production to achieve effective and legitimate solutions to the decline and loss of biocultural diversity.

To achieve all this, a profound and urgent transformation of research and research institutions is needed to recognise ILK and then to develop the skills required to see, engage with and understand ILK. The persistence of research *on* IPLC compared to research *with* IPLC (Koster et al. 2012) prompted us to engage in a thought experiment involving a non-Indigenous researcher (sometimes called an external researcher by Indigenous communities) conducting empirical research. We named this scenario "the ILK as a door metaphor".

### Indigenous and local knowledge as a door to a pathway to inclusion

To conclude our discussion, we reflect on our understanding of the value of ILK in biodiversity and conservation research and beyond. Scientists working with tropical forest fauna should better recognise and engage with the incredible wealth of knowledge and multiple ways of understanding the complexity of tropical forest ecosystems, as well as how people are an integral component of these. Nonetheless, a substantial limitation that is slowly changing is the disciplinary boundaries set by research and educational institutions. Very few ecologists and biologists receive training in social sciences, such as anthropology or environmental sociology, and may be, what we call, ILK blind. ILK blindness is a very broad term that can be addressed through academic training that policy makers, natural scientists and ecologists could and should undertake to broaden their understanding of ILK. This would improve research and make science, as well as conservation, more inclusive and account for biocultural diversity and the profound social–ecological interactions.

**Fig. 7:**
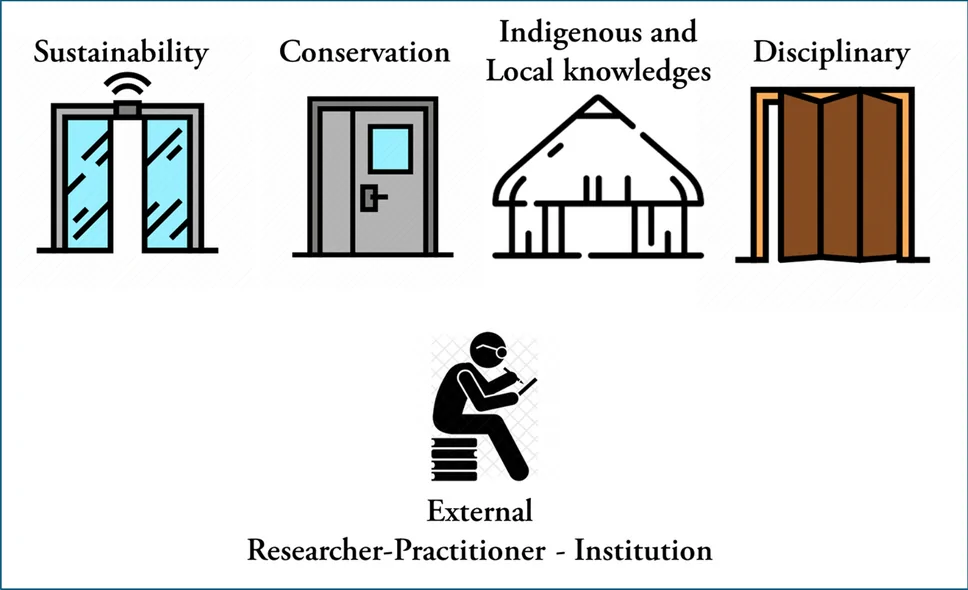
Door metaphor for researchers entering and studying Forest Fauna with ILK and IPLC, among other doors

Different scenarios related to individual researcher experiences, institutional constraints or project characteristics can be better addressed using what we propose here as the metaphor of: ILK “as a Door” to IPLC knowledge systems. This metaphor can serve to understand a variety of research/researcher scenarios, with different aims and objectives, that engage, whether fully or partially, with ILK. We view the door as an entry point into various epistemologies, ontologies, human–nature relationships, values and knowledges about, in this instance, tropical forest fauna and its management. The following are our theorisations to this effect:

1. Researchers cannot see the ILK Door (e.g. ILK blind scenario), only seeing the other most common and visible doors, such as conservation, sustainability or its own disciplinary door of training (Fig. 7).
2. Researchers see the Door but do not describe it (e.g. project constraints)
3. Researchers see and move towards the Door but cannot access (context and consent constraints)
4. Researchers see the door, move towards, enter or are invited to enter the door and describe the shared understanding (willing and prepared)
5. Researchers already knew how to see the door, already knew it and have been inside before and visit or live inside (habituated)
6. Researchers have moved beyond merely seeing a door and moving between contexts, using the door's label to share with others without experiencing it (integrated).

ILK can serve as a door to a deeper understanding of IPLC ways of being and relating to each other and the world, and, in turn, tropical forest fauna and their management. Following this door metaphor, we found that while most studies saw the door and referred to it as ILK, they did not open it. Others did not appear to see the door or name it. Only a handful of studies saw, named and actually opened the door to pass through it. Our analysis of whether there are any observable characteristics that contribute to determining how authors have engaged with ILK can be of use for diverse scientific institutions and researchers to reflect on their own interest, preparedness and willingness to enter through this ILK door. As to the why—we can only speculate—but based on our own experiences and insights it is probably a combination of time, money, training and a traditional education in biology/ecology that largely excludes local people from scientific research processes.

Authors such as Bartlett et al. (2012), Hogue (2019) and Latuklippe and Klenk (2020) have come to similar conclusions and highlight that we first must bring attention to the “Two-Eyed Seeing” principle to weave Indigenous and mainstream knowledges together. The Two-Eyed Seeing principle refers to learning to see with one eye using the strengths of ILK and ways of knowing, and from the other eye, with the strengths of Western (mainstream) ways of knowing. Yet, and most importantly, learning to use both eyes together for truly informed vision (Bartlett et al. 2012; Hogue 2019). Second, the “Making a Room and Moving Over” principle is a critical position to knowledge co-production together with ILK, explaining why co-production scholars should move from merely “seeking to better integrate” ILK into Western science, but instead make way for IPLC research leadership (Latulippe and Klenk 2020). Third, we aligned with the research framework **“**Moving from research *on*, to research *with* and *for* Indigenous communities”, arguing that researchers need to move away from methods that perpetuate traditional ways of working *on* Indigenous and Local Communities to methods that work *with* and *for* IPLC (Koster et al. 2012).

In this sense, to continue this evolution from *on* towards *with* and *for* we need to become proficient at seeing with two eyes where ILK is an equal door through which we must go to ensure that equal knowledge authority is given to IPLC.

## Conclusion

Based on our review, we see a need for a future research agenda on ILK of tropical forest fauna. There are numerous important benefits in committing to greater reflexivity and advancements in research that involve collaboration rather than isolation. We acknowledge that gaining a deeper insight into the significance of ILK regarding forest fauna requires an inter- and transdisciplinary approach. Actionable knowledge can be fostered at every level, even within specific fields of study. A substantial shift towards greater inclusion can enhance not just our comprehension but also the management of tropical forest social–ecological systems. This will, we argue, ultimately empower IPLC in their fight for greater autonomy and self-determination through data collection to the access and dissemination of research findings. Deeper self-reflection on the historical patterns of knowledge system asymmetries and their connection to individual perspectives and ideologies is indeed needed for the transformation towards knowledge inclusion, plurality and justice. The diversity of labels and names currently referred to as ILK is indicative of the broad efforts for IPLC inclusion, one that is also confronted by the evident invisibility of ILK to many other researchers and institutions. Recognition of ILK value is closely related to respectful naming as a first step to consider respectful investigation and dialogue with IPLC. We consider this connection of practitioners and epistemic revision to be of fundamental importance, one that is closely related and addressed by decolonial studies. One positive development is the increasing number of publications that include non-Indigenous and Indigenous scholars referring to social-ecological paradigm shifts and methodologies that enrich our understanding of forest fauna, local management and resource use. More awareness and training of researchers can influence their willingness to dialogue with ILK that promotes reflexivity can help to ensure that the vast knowledge of IPLC, as well as their autonomy and right to self-determination, is respected and taken seriously. While only one concept of many, we identify that “weaving of knowledge” concept and practice is a crucial vehicle for working towards deep and sustained co-production in research.

As the pressure on tropical forest biodiversity continues to rise, pushing many populations and entire species towards extinction, we also need to recognise how wide-ranging environmental changes are intertwined with and are the result of socio-economic and political changes. Thus, the ongoing loss of biodiversity is in fact a loss of biocultural diversity, as it leads to the erosion and loss of ILK and the loss of local languages as living libraries. The invaluable knowledge of IPLC is not only a critical source of information revealing the intricacies of local social–ecological systems, but can also help in the struggle to safeguard tropical forest ecosystems from a biocultural perspective. Moreover, from a procedural and rights-based approach, IPLC have an inherent right to participate in research about them or that affects them, because they are rightful users and legal owners of territories located in tropical forest areas. From an instrumental perspective, we need to document, include, and centre ILK because it is a door that, if we dare to pass through, can provide us with an incredible source of information that, in turn, can promote a pathway to a more meaningful, inclusive and effective conservation of tropical forests as social–ecological systems.

## Supplementary Information

Below is the link to the electronic supplementary material.

Supplementary file1 (PDF 409 KB)
