# Supplementary material for: Indigenous and local knowledge inclusion in forest fauna research: A systematic review in the tropics
Source: Ambio. 2026 Apr 24;55(10):2377–94. doi: 10.1007/s13280-026-02378-y (PMC13554006; doi:10.1007/s13280-026-02378-y)
Supplement: Supplementary file 1 [file 13280_2026_2378_MOESM1_ESM.pdf]

Ambio

Supplementary Information

This supplementary information has not been peer reviewed.

Title:

Indigenous and Local Knowledges inclusion in Forest Fauna research - A Systematic Review in the Tropics

Supplementary Material (S)

S1

Stage 1. Evolution of the search string in the exploratory stage.
Figure – Presents the first stage of this review, where we conducted an exploratory search using strings 1-4 before arriving at the Stage 2 Search String.

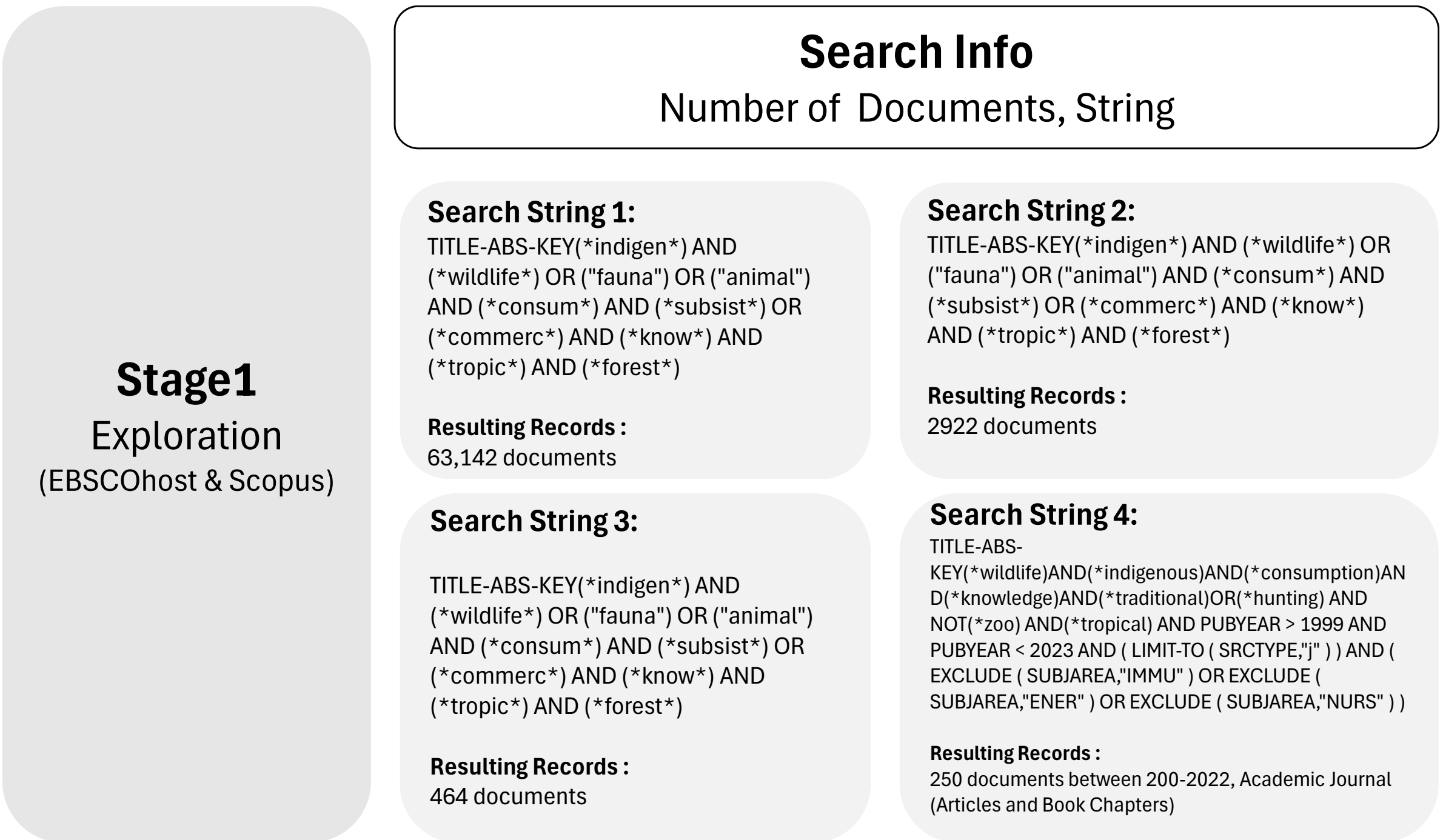

S2

Categories and Labels

Table 1 – Present the seven categories and 52 labels uploaded in Sysrev© 2025 to gather information from the 62 revised articles.

	Category of Info	Num	Label Name
	I. Inclusion Criteria	I	Inclusion
1	(1.0) Document Info	1.1	First Author Name
2		1.2	Year of Publication
3		1.3	Country of Institution Affiliation of First Author
4		1.4	Name of Funding Origins
5		1.5	Academia Program Related
6		1.6	Discipline/Field of Study
7		1.7	Thematics or Topics Mentioned
8		1.8	List the key words
9		1.9	Type of Document "Purpose"
10	(2.0) Region, Community and Ecosystem	2.1	Country of study
11		2.2	Name of Region - Site
12		2.3	Name of ethnic/tribe/peasant group
13		2.4	Type of ecosystem
14	(3.0) METHODS	3.1	Duration of Study (months)
15		3.2	Research Question
16		3.3	Research Methods/Approach
17		3.4	Methods Category
18		3.5	Degree of Local Participation
19	(4.0) Village Community Info	4.1	Size of the Community
20		4.2	Number of Families
21		4.3	Distance/conection to Urban Centers
22		4.4	Territory/Land Category
23		4.5	Livelihood activities of target groups
24	(5.0) TEK INFO (Interactions	5.1.1	TEK Defined (Yes / No)
25		5.1.2	TEK Definition
26		5.1.4	Authors/Doc Position towards TEK (Relation to Discipline or Theory)
27		5.1.5	TEK Practice Description
28		5.1.6	TEK Prohibition Description
29		5.1.7	TEK Rules n Norms
30		5.1.8	TEK Believes Description
31		5.1.9	TEK Vitality Status
32		5.1.10	TEK Leadership
33		5.1.11	TEK Holders
34		5.1.12	TEK Drivers of Loss of
35		5.1.13	TEK Conflicts
36	(6.0) WildLife Use Info	6.1	Type of use of wild fauna
37		6.2	Wildlife Main Purpose of Use
38		6.3	Wild Fauna Frequency of Use
39		6.4	Wild Fauna Quantities when used
40	(7.0) Fauna Info	7.1.1	Wildlife Local Names Provided
41		7.1.2	IUCN Categories
42		7.1.3	Special Wild Fauna Category
43		7.1.4	Scientific Names Provided
44		7.1.5	Special Wildlife Scientific names
45		7.1.6	Wild Fauna No. Species Involved
46		7.1.7	Birds No. Species Involved
47		7.1.8	Mammals No. Species Involved
48		7.1.9	Reptiles No. Species Involved
49		7.1.10	Amphibians No. Species Involved
50		7.1.11	Invertebrates No. Species Involved
51		7.1.12	Other Wilife Category No.

S3

Table 2. List of the frequency of the 42 reported combinations of Forest Fauna Uses.

Type of use of wild fauna	Fauna Uses reported in 62 docs
Edible	13
Edible, Commerce	6
Edible, Medicine, Ceremonial	4
Edible, Medicine	2
Edible, Ceremonial	2
Edible, Medicine, Commerce	2
Edible, Medicine, Ornaments, Ceremonial, Commerce, Other	1
Edible, Tools, Ornaments, Ceremonial, Indicator of, Companion, Medicine	1
Edible, Medicine, Tools, Ornaments, Ceremonia, Companion, Commerce, Indicator of, Tourism, Other	1
Edible, Ceremonial, Commerce, Tourism, Other	1
Medicine, Edible, Ceremonial, Indicator of	1
Commerce, Edible, Medicine	1
Edible, Medicine, Ornaments, Tools, Commerce, Ceremonial	1
Edible, Commerce Other	1
Edible, Tools, Commerce	1
Edible, Commerce, Ceremonial	1
Indicator of	1
Edible, Companion, Commerce	1
Edible, Medicine, Ornaments	1
Edible, Companion, Commerce, Ornaments, Medicine	1
Edible, Medicine, Ornaments, Ceremonial, Companion, Indicator of, Tourism	1
Edible, Ceremonial, Commerce	1
Edible, Medicine, Tools, Ceremonial	1
Edible, Ceremonial, Commerce, Medicine	1
Edible, Ornaments, Medicine, Ceremonial	1
Edible, Medicine, Ceremonial, Companion, Ornaments	1
Edible, Tools, Medicine, Ceremonial, Commerce, Tourism, Other	1
Medicine, Edible, Commerce	1
Edible. Commerce, Medicine. Tourism, Ceremonial, Ornaments	1
Medicine, Edible, Other	1
Indicator of, Ceremonial	1
Ornaments, Ceremonial	1
Edible, Ceremonial, Commerce, Medicine, Ornaments	1
Commerce, Edible	1
NOT MENTIONED	1
Edible, Medicine, Commerce, Companion	1
Tourism, Indicator of	1
Edible, Medicine, Commerce, Companion.	1
Edible, Medicine, Commerce, Indicator of, Ornaments	1
**Grand Total**	**62**

S4

Table 3. List and count of the ten main TEK categories that the authors identified in 25 of the 62 documents revised.

No.	Knowledge Category	Abrev.	Counts
1	Traditional Ecological Knowledge +	TEK+	9
2	Experiential/Traditional Knowledge	ETK	1
3	Strategic Relational Symbiosis	SRS	1
4	Local Traditional Knowledge/Indigenous Knowledge	LTK+	5
5	Local Ecological Knowledge	LEK	2
6	Ecological Knowledge	EK	1
7	Local Environmental Knowledge	LEn-K	1
8	Cultural Traditions Nature Interdependent	Descriptive	3
9	Ancestral Knowledge	AK	1
10	Local Wisdom-LK	LW-LK	1
		**Total #** documents defining knowledge	25

S5

Table 4. Direct citation describing methods or community participation that exemplify each of the five degrees of participation categorised by the authors.

**Participation Degree** (meaning)	**Methods - Description**	**Example of Reference**
**Shared decisions** (community is partner in research process)	"*The development of the strategy emphasized the involvement of hunters and other traditional authorities, and the process involved adapting and testing existing local wildlife management systems*" p.38. in Akumsi, 2003.<br><br>*"This paper is not intended as an objective, empirical investigation of Solega people's attitudes toward, and practices regarding, conflict with wild animals. Instead, we present the following information with a two-fold aim. The first is to give a voice to Solega people, as indigenous voices and points of view are often absent from discourses surrounding endangered wildlife, invasive species, and general biodiversity conservation (Barbour and Schlesinger, 2012; Bhattacharyya and Larson, 2014)"*.<br>p.4. in Agnihotri et al 2021.<br><br>"*When indigenous people are mentioned in these discourses, they tend to be portrayed in familiar, conventionalised ways (Reo et al., 2017b). Moreover, even researchers who are sympathetic to indigenous concerns may find it appropriate to suppress the more emic aspects of their study in the name of objectivity, quantifiability, scientific rigour and neutrality (Datta, 2018)"*.<br>p.4. in Agnihotri et al 2021.<br><br>*"The second aim is to demonstrate to non-indigenous stakeholders (such as forestry officials, conservation scientists, wildlife enthusiasts in the general public) that core elements of Solega culture predispose the community to playing a key role in conservation efforts, should they be afforded the opportunity to do so"*.<br>p.4. in Agnihotri et al 2021.	(Akumsi, 2003; Agnihotri *et al.*, 2021)
**Involved** (researchers work directly with community)	Open Interviews, Participant Observation, with key informants<br><br>"*The objective of this work was to identify the species of edible invertebrates by ethnicities Yanomami and Yekuana from the Alto Orinoco*"<br>p.319.<br><br>*"Most of the informants understood Spanish or were translated into their indigenous language through a local translator"*.<br>p.319.	Venezuela (Araújo & Beserra, 2007)
**Consulted** (input is obtained from the community)	*"Here, our aim was to assess the population status of rusa deer, and to investigate the extent of hunting practices on this mammal in West Papua…We conducted camera trapping and line transect surveys simultaneously to estimate…We also interviewed hunters (n = 134), informants (n=9) and households (n=91) to assess hunting patterns and socioeconomic importance of rusa deer across 15 districts of the Tambrauw regency."* (Pangau-Adam *et al.*, 2021, p.1).	(Pangau-Adam *et al.*, 2021)

**Informed** (information is provided to community)	"*To assess local knowledge and uses of pangolins in the study area, we used a semi-structured interview questionnaire comprised of both open- and closed-ended questions*" p.4.<br><br>"*semi-structured interview questionnaire inferential analyses Binomial generalized linear models (GLM) with a logit link function were built for dependent variables having two outcomes such as whether the respondent recognised the species or had seen it before (Yes/No)*". p.4.	(Mouafo *et al.*, 2021)
**Not Apply** (Systematic Review)	"*We reviewed 81 years (1937–2018) of campesino hunting scholarship to determine the state of knowledge about campesino hunting in Latin America*." p.339.	(Petriello & Stronza, 2020)

S6

List of articles included in the Systematic Review (62 documents)
with **Review Document Number in bold**, citation and links to Sysrev platform and document link

Review Document No. (RDN)	Document as loaded in SYSREV	Citation	Sysrev Link	DOI / Link
**RDNo.1**	Doc91. Invertebrados Consumidos Yanomami Vezuale_ Araujo_2006..pdf	Araújo Y, Beserra P. Diversidad en invertebrados consumidos por las etnias Yanomami y Yekuana del Alto Orinico, Venezuela. Interciencia. 2007;32(5):318-23.	https://www.sysrev.com/p/139927/article/96359294	http://www.redalyc.org/articulo.oa?id=33932505
**RDNo.2**	Doc94. Faunal Use Mro Tribe Bangladesh_Chowdry_2010..pdf	Chowdhury, M. S. H., Halim, Md. A., Miah, Md. D., Muhammed, N., & Koike, M. (2007). Research Communication: Biodiversity use through harvesting faunal resources from forests by the Mro tribe in the Chittagong Hill Tracts, Bangladesh. International Journal of Biodiversity Science & Management, 3(1), 56–62. https://doi.org/10.1080/17451590709618162	https://www.sysrev.com/p/139927/article/96359297	https://doi.org/10.1080/17451590709618162
**RDNo.3**	Doc97. Community participation Wildlife Manag_Akumsi_2003..pdf	Akumsi, A. (2003). Community participation in wildlife management: the Mount Cameroon experience. Unasylva, 54(3), 37-42.	https://www.sysrev.com/p/139927/article/96359300	https://www.fao.org/4/y5189e/y5189e06.pdf
**RDNo.4**	Doc103. Importance of leaf-litter feeding invertebrates Amazon_ Paoletti Dufour_2000..pdf	Paoletti, M. G., Dufour, D. L., Cerda, H., Torres, F., Pizzoferrato, L., & Pimentel, D. (2000). The importance of leaf-and litter-feeding invertebrates as sources of animal protein for the Amazonian Amerindians. Proceedings of the Royal Society of London. Series B: Biological Sciences, 267(1459), 2247-2252.	https://www.sysrev.com/p/139927/article/96359306	doi10.1098/rspb.2000.1275
**RDNo.5**	Doc1. Knowledge of Wildlife. Evelyn Pina.2022..pdf	Piña-Covarrubias, E., Chávez, C., & Doncaster, C. P. (2022). Knowledge of wildlife, hunting, and human-felid interactions in Maya forest communities of the northern yucatán peninsula, Mexico. Human Ecology, 50(6), 1035-1045.	https://www.sysrev.com/p/139927/article/96359313	https://doi.org/10.1007/s10745-022-00363-z
**RDNo.6**	Doc2. Hunting & Wildlife Brasil.Jose Barbosa. 2022..pdf	Barbosa, J. A. A., Aguiar, J. O., & da Nóbrega Alves, R. R. (2022). Hunting and wildlife use in protected areas of the Atlantic rainforest, northeastern Brazil. Desenvolvimento e Meio Ambiente, 60.	https://www.sysrev.com/p/139927/article/96359314	DOI: 10.5380/dma.v60i0.74388 e-ISSN 2176-9109
**RDNo.7**	Doc3. Sustainability Game Hunting. R Sampaio. 2022..pdf	Sampaio, R., Morato, R. G., Abrahams, M. I., Peres, C. A., & Chiarello, A. G. (2022). Physical geography trumps legal protection in driving the perceived sustainability of game hunting in Amazonian local communities. Journal for Nature Conservation, 67, 126175.	https://www.sysrev.com/p/139927/article/96359315	https://doi.org/10.1016/j.jnc.2022.126175
**RDNo.8**	Doc4. Food Nutrition Colombia. Jefferson Asprilla.2021..pdf	Asprilla-Perea, J., Díaz-Puente, J. M., & Martín-Fernández, S. (2021). Estimating the potential of wild foods for nutrition and food security planning in tropical areas: Experimentation with a method in Northwestern Colombia. Ambio, 1-17.	https://www.sysrev.com/u/14052/p/139927/article/96359316	https://doi.org/10.1007/s13280-021-01624-9
**RDNo.9**	Doc5. Congruence of LEK_2021..pdf	Braga-Pereira, F., Morcatty, T. Q., El Bizri, H. R., Tavares, A. S., Mere-Roncal, C., González-Crespo, C., ... & Mayor, P. (2022). Congruence of local ecological knowledge (LEK)-based methods and line-transect surveys in estimating wildlife abundance in tropical forests. Methods in Ecology and Evolution, 13(3), 743-756.	https://www.sysrev.com/p/139927/article/96359317	DOI: 10.1111/2041-210X.13773
**RDNo.10**	Doc6. Farmers Perspective Orangutan. Harahap_2022..pdf	Harahap, H. A., & Ridwan, E. (2022, December). Assessing the local farmer's perspective on Tapanuli Orangutan in Agroforest Landscape of Batang Toru Ecosystem, Indonesia. In IOP Conference Series: Earth and Environmental Science (Vol. 1115, No. 1, p. 012021). IOP Publishing.	https://www.sysrev.com/u/14052/p/139927/article/96359318	DOI 10.1088/1755-1315/1115/1/012021

**RDNo.11**	Doc7. Farmers persepective Bat EcosServ. Harahap_2022..pdf	HARAHAP, H. A., & YONARIZA, Y. (2022). Assessing local farmer's perspectives on the role of bat in providing ecosystem services in the Batang Toru ecosystem, North Sumatra, Indonesia. Biodiversitas Journal of Biological Diversity, 23(8).	https://www.sysrev.com/u/14052/p/139927/article/96359319	DOI: 10.13057/biodiv/d230857
**RDNo.12**	Doc8. Patterns Wildlife Hunting Amaz. Silva_ 2022..pdf	da Silva, A. B., Pereyra, P. E., El Bizri, H. R., Souto, W. M., & Barboza, R. S. L. (2022). Patterns of wildlife hunting and trade by local communities in eastern Amazonian floodplains. Ethnobiology and Conservation, 11.	https://www.sysrev.com/p/139927/article/96359320	doi:10.15451/ec2022-07-11.16-1-19
**RDNo.13**	Doc9. Cervus timorensis unting WPapua_Margaretha Pangau_2021..pdf	Pangau-Adam, M., Flassy, M., Trei, J. N., Waltert, M., & Soofi, M. (2022). The role of the introduced rusa deer Cervus timorensis for wildlife hunting in West Papua, Indonesia. Ecological Solutions and Evidence, 3(1), e12118.	https://www.sysrev.com/u/14052/p/139927/article/96359321	https://doi.org/10.1002/2688-8319.12118
**RDNo.14**	Doc10. Hunting Wild WPapua_Saremay Sawaki_ 2022..pdf	Pattiselanno, F., & Krockenberger, A. (2021). Road development and Indigenous hunting in Tanah Papua: Connecting the facts for future wildlife conservation agendas. Forest and Society, 5(1), 181-189.	https://www.sysrev.com/p/139927/article/96359322	DOI: 10.13057/biodiv/d230519
**RDNo.15**	Doc13. Networks EcoKnoweldge_L.Ong_2021..pdf	Ong, L., Campos-Arceiz, A., Loke, V. P., Pura, P. B., Tunil, C. M. T. B., Din, H. S. A. L., ... & McConkey, K. R. (2021). Building ecological networks with local ecological knowledge in hyper-diverse and logistically challenging ecosystems. Methods in Ecology and Evolution, 12(10), 2042-2053.	https://www.sysrev.com/p/139927/article/96359324	DOI: 10.1111/2041-210X.13685
**RDNo.16**	Doc15. Camera Traps Community Knowledge Pangolin_ Vikram Aditya_2019..pdf	Aditya, V., Komanduri, K. P., Subhedar, R., & Ganesh, T. (2021). Integrating camera traps and community knowledge to assess the status of the Indian pangolin Manis crassicaudata in the Eastern Ghats, India. Oryx, 55(5), 677-683.	https://www.sysrev.com/p/139927/article/96359326	https://doi.org/10.1017/S0030605319001303
**RDNo.17**	Doc16. Ethnornitho Knowledge_J.Iskandar_ 2021..pdf	ISKANDAR, J., HUSODO, T., WULANDARI, I., MEGANTARA, E. N., PARTASASMITA, R., & SHANIDA, S. S. (2021). Bird diversity and ethno-ornithological knowledge of local people in Ciletuh-Palabuhanratu Geopark, Sukabumi, West Java, Indonesia. Biodiversitas: Journal of Biological Diversity, 22(8).	https://www.sysrev.com/u/14052/p/139927/article/96359327	DOI: 10.13057/biodiv/d220838
**RDNo.18**	Doc19. Community Knoweldge-Adapt-Cimate_Matos_ 2021..pdf	Matos, A., Barraza, L., & Ruiz-Mallén, I. (2021). Linking conservation, community knowledge, and adaptation to extreme climatic events: a case study in Gorongosa National Park, Mozambique. Sustainability, 13(11), 6478.	https://www.sysrev.com/p/139927/article/96359330	https://doi.org/10.3390/su13116478
**RDNo.19**	Doc20. Anthropogenic Perception noct Primate_Fominka_2020..pdf	Fominka, N. T., Oliveira, H. F., Taboue, G. C. T., Luma, F. E., Robinson, C. A., & Fokam, E. B. (2021). Conserving the forgotten: New insights from a Central African biodiversity hotspot on the anthropogenic perception of nocturnal primates (Mammalia: Strepsirrhini). Primates, 62, 537-546.	https://www.sysrev.com/p/139927/article/96359331	https://doi.org/10.1007/s10329-021-00898-7
**RDNo.20**	Doc23. Biocultural Lens Bat Interaction_Rocha_2021..pdf	Rocha R, Fernández-Llamazares Á, López-Baucells A, Andriamitandrina SF, Andriatafika ZE, Temba EM, et al. Human-bat interactions in rural Southwestern Madagascar through a biocultural lens. Journal of Ethnobiology. 2021;41(1):53-69.	https://www.sysrev.com/p/139927/article/96359334	https://doi.org/10.2993/0278-0771-41.1.53
**RDNo.21**	Doc24. Conocimiento Ancestral Nontimber Prod_ Vivanco_2021..pdf	Maza, D., Abad, S., Malagón, O., & Armijos, C. (2021). Productos forestales no maderables de la comunidad El Tundo: Un remanente boscoso de biodiversidad y conocimiento ancestral del sur del Ecuador. Rev. Bionatura, 6, 2161-2174.	https://www.sysrev.com/u/14052/p/139927/article/96359335	DOI. 10.21931/RB/2021.06.04.5
**RDNo.22**	Doc25. Tiger Termite Hill WL Perceptions_Agnihotri_2021..pdf	Agnihotri, S., Madegowda, C., & Si, A. (2021). Tiger becomes termite hill: Soliga/Solega perceptions of wildlife interactions and ecological change. Frontiers in Conservation Science, 2, 691900	https://www.sysrev.com/p/139927/article/96359336	doi: 10.3389/fcosc.2021.691900

**RDNo.23**	Doc26. LocalKnowledge Pangolin_Mouafo_2021..pdf	Mouafo, A. D., Ingram, D. J., Tegang Pagning, R., Nfor Ngwayi, I. C., & Mayaka, T. B. (2021). Local knowledge and use of pangolins by culturally diverse communities in the forest-savannah transition area of Cameroon. Tropical Conservation Science, 14, 19400829211028138.	https://www.sysrev.com/u/14052/p/139927/article/96359337	DOI: 10.1177/19400829211028138
**RDNo.24**	Doc27. Roadsand Indigenous Hunitng_Pattiselano_2021..pdf	Pattiselanno, F., & Krockenberger, A. (2021). Road development and Indigenous hunting in Tanah Papua: Connecting the facts for future wildlife conservation agendas. Forest and Society, 5(1), 181-189.	https://www.sysrev.com/p/139927/article/96359335	https://journal.unhas.ac.id/index.php/fs/article/view/12528
**RDNo.25**	Doc28. Etnozoologico Valor Nutricional Venado_GarciaFlores_2021..pdf	García-Flores, A., Valle-Marquina, R., Monroy-Martínez, R., Sánchez, S. B., & Pino-Moreno, J. M. (2021). Estudio etnozoológico y valor nutricional del venado cola blanca Odocoileus virginianus en la comunidad de Pitzotlán, Tepalcingo, Morelos, México. Caldasia, 43(1), 105-116.	https://www.sysrev.com/p/139927/article/96359339	https://www.jstor.org/stable/10.2307/48646890
**RDNo.26**	Doc29. Zoonotic risk Human Wildlife_ Namusisi_2021..pdf	Namusisi, S., Mahero, M., Travis, D., Pelican, K., Robertson, C., & Mugisha, L. (2021). A descriptive study of zoonotic disease risk at the human-wildlife interface in a biodiversity hot spot in South Western Uganda. PLoS neglected tropical diseases, 15(1), e0008633.	https://www.sysrev.com/p/139927/article/96359340	https://doi.org/10.1371/journal.pntd.0008633
**RDNo.27**	Doc30. Sociocultural Significance Mineral Licks_Gilmore_2020..pdf	Gilmore, M. P., Griffiths, B. M., & Bowler, M. (2020). The socio-cultural significance of mineral licks to the Maijuna of the Peruvian Amazon: implications for the sustainable management of hunting. Journal of Ethnobiology and Ethnomedicine, 16, 1-10.	https://www.sysrev.com/p/139927/article/96359341	https://doi.org/10.1186/s13002-020-00412-1
**RDNo.28**	Doc31. Local Ecological Knowledge wild resources impoverishment_Coomes_2020..pdf	Coomes, O. T., Takasaki, Y., & Abizaid, C. (2020). Impoverishment of local wild resources in western Amazonia: a large-scale community survey of local ecological knowledge. Environmental Research Letters, 15(7), 074016.	https://www.sysrev.com/p/139927/article/96359342	DOI 10.1088/1748-9326/ab83ad
**RDNo.29**	Doc32. People and Birds Interactions_Romero-Bautista_2020..pdf	Romero-Bautista, Y. A., Moreno-Calles, A. I., Alvarado-Ramos, F., Reyes Castillo, M., & Casas, A. (2020). Environmental interactions between people and birds in semiarid lands of the Zapotitlán Valley, Central Mexico. Journal of Ethnobiology and Ethnomedicine, 16, 1-14.	https://www.sysrev.com/u/14052/p/139927/article/96359343	https://doi.org/10.1186/s13002-020-00385-1
**RDNo.30**	Doc33. Ceremonial use PesquetParrot_ Nugi_2019..pdf	Grace Nugi & Nathan Whitmore (2020) More dead than alive: harvest for ceremonial headdresses threatens Pesquet's Parrot in Papua New Guinea, Emu - Austral	https://www.sysrev.com/p/139927/article/96359344	https://doi.org/10.1080/01584197.2019.1676162
**RDNo.31**	Doc34. Campesino Hunting_Petriello_2019..pdf	Petriello, M. A., & Stronza, A. L. (2020). Campesino hunting and conservation in Latin America. Conservation Biology, 34(2), 338-353.	https://www.sysrev.com/p/139927/article/96359345	http://www.ethnobiomed.com/content/5/1/36
**RDNo.32**	Doc35. Indigenous Hunting Practices_Loke_2020..pdf	Ornithology, 120:2, 156-161,	https://www.sysrev.com/u/14052/p/139927/article/96359346	https://doi.org/10.1016/j.gecco.2019.e00815
**RDNo.33**	Doc38. Ethic Knowledge Heterogeneity consumption_Kekenou_2020..pdf	Kekeunou, S., Laïda, P. S., Achu-Loh, M. B., Tchouamou, C. D., Simo-Guiadem, L., Pieme, C. A., ... & Tamesse, J. L. (2020). Ethnic heterogeneity of knowledge on Zonocerus variegatus and reasons for consumption and non-consumption in the southern part of Cameroon. Journal of Insects as Food and Feed, 6(3), 273-284.	https://www.sysrev.com/u/14052/p/139927/article/96359349	DOI 10.3920/JIFF2019.0035
**RDNo.34**	Doc39. Baby Pangolins on myplate_Volpato_2020..pdf	Volpato, G., Fontefrancesco, M. F., Gruppuso, P., Zocchi, D. M., & Pieroni, A. (2020). Baby pangolins on my plate: possible lessons to learn from the COVID-19 pandemic. Journal of Ethnobiology and Ethnomedicine, 16, 1-12.	https://www.sysrev.com/p/139927/article/96359350	https://doi.org/10.1186/s13002-020-00366-4
**RDNo.35**	Doc41. Cuicatec Traditional Knowledge Fauna_Solis_2019..pdf	Solís, L., & Casas, A. (2019). Cuicatec ethnozoology: traditional knowledge, use, and management of fauna by people of San Lorenzo Pápalo, Oaxaca, Mexico. *Journal of Ethnobiology and Ethnomedicine*, *15*(1), 58.	https://www.sysrev.com/p/139927/article/96359352	https://doi.org/10.1186/s13002-019-0340-1

**RDNo.36**	Doc42. Low Intensity Hunting impact Game species_Hallet_2019..pdf	Hallett, M. T., Kinahan, A. A., McGregor, R., Baggallay, T., Babb, T., Barnabus, H., ... & Bankovich, B. A. (2019). Impact of low-intensity hunting on game species in and around the Kanuku Mountains Protected Area, Guyana. Frontiers in Ecology and Evolution, 7, 412.	https://www.sysrev.com/u/14052/p/139927/article/96359353	https://doi.org/10.3389/fevo.2019.00412
**RDNo.37**	Doc43. Understanding Ibans TEK on Hornbills_Franco_2019..pdf	Franco, F. M., & Minggu, M. J. (2019). When the seeds sprout, the hornbills hatch: understanding the traditional ecological knowledge of the Ibans of Brunei Darussalam on hornbills. Journal of ethnobiology and ethnomedicine, 15, 1-14.	https://www.sysrev.com/u/14052/p/139927/article/96359354	https://doi.org/10.1186/s13002-019-0325-0
**RDNo.38**	Doc44. Conservation based in Indigenous Knowledge_Mavhura_2019..pdf	Mavhura, E., & Mushure, S. (2019). Forest and wildlife resource-conservation efforts based on indigenous knowledge: The case of Nharira community in Chikomba district, Zimbabwe. Forest Policy and Economics, 105, 83-90.	https://www.sysrev.com/p/139927/article/96359355	https://doi.org/10.1016/j.forpol.2019.05.019
**RDNo.39**	Doc46. Mammal Subistence Hunting_Mesquita_2018..pdf	Pires Mesquita, G., Domingo Rodríguez-Teijeiro, J., & Nascimento Barreto, L. (2018). Patterns of mammal subsistence hunting in eastern Amazon, Brazil. Wildlife Society Bulletin, 42(2), 272-283.	https://www.sysrev.com/p/139927/article/96359357	DOI: 10.1002/wsb.873
**RDNo.40**	Doc47. Paca breeding seasonality_ElBizri_2018..pdf	El Bizri, H. R., Fa, J. E., Bowler, M., Valsecchi, J., Bodmer, R., & Mayor, P. (2018). Breeding seasonality in the lowland paca (Cuniculus paca) in Amazonia: interactions with rainfall, fruiting, and sustainable hunting. Journal of Mammalogy, 99(5), 1101-1111.	https://www.sysrev.com/p/139927/article/96359358	DOI:10.1093/jmammal/gyy102
**RDNo.41**	Doc48. Material Culture Jarawa_Sahrief_2018..pdf	Sharief, M. U., & Panda, S. P. (2018). Material culture of the ethnic aboriginal Jarawa folk in Andaman Islands, India. Genetic Resources and Crop Evolution, 65, 1369-1384.	https://www.sysrev.com/u/14052/p/139927/article/96359359	https://doi.org/10.1007/s10722-018-0620-y
**RDNo.42**	Doc50. Local Views Private vs Community Conserv_Zafra-Calvo_2018..pdf	Zafra-Calvo, N., & Moreno-Peñaranda, R. (2018). Exploring local people's views on the livelihood impacts of privately versus community managed conservation strategies in the Ruvuma landscape of North Mozambique-South Tanzania. Journal of Environmental Management, 206, 853-862.	https://www.sysrev.com/p/139927/article/96359361	https://doi.org/10.1016/j.jenvman.2017.11.065
**RDNo.43**	Doc51. Sacred Landscapes rigths world_Bodra_2019..pdf	Hembrom, G. B. (2018). Sacred landscape, modes of subsistence and Adivasi rights in the globalised world. In Globalisation, Environment and Social Justice (pp. 261-300). Routledge India.	https://www.sysrev.com/u/14052/p/139927/article/96359362	https://doi.org/10.4324/9780429455766
**RDNo.44**	Doc52. Mamif&Aves usadas pobladores_Vela_2017..pdf	Vela Alvarado, J. W., Rivas, M., Fernández, V., & Clavo Peralta, M. (2017). Mamíferos y aves silvestres usados por los pobladores de la cuenca del río Abujao (Ucayali, Perú). Revista peruana de biología, 24(3), 263-272.	https://www.sysrev.com/u/14052/p/139927/article/96359363	https://doi.org/10.15381/rpb.v24i3.13907
**RDNo.45**	Doc53. Percepcion Rural d Uso Avifauna_Barbaran_2017..pdf	Barbarán, F. (2017). Percepción y uso de la avifauna en ecosistemas rurales de Salta, Jujuy y sur de Bolivia. El hornero, 32(1), 63-71.	https://www.sysrev.com/p/139927/article/96359364	https://www.scielo.org.ar/pdf/hornero/v32n1/v32n1a07.pdf
**RDNo.46**	Doc55. Regaining Traditional Use WL food security_Gonzales-Marin_2017..pdf	González-Marín, R. M., Moreno-Casasola, P., Castro-Luna, A. A., & Castillo, A. (2017). Regaining the traditional use of wildlife in wetlands on the coastal plain of Veracruz, Mexico: ensuring food security in the face of global climate change. Regional Environmental Change, 17, 1343-1354.	https://www.sysrev.com/u/14052/p/139927/article/96359366	DOI 10.1007/s10113-016-0955-x
**RDNo.47**	Doc60. Wildlife Use Indigenous WGhats_Kanagavel_2016..pdf	Conservation implications of wildlife utilization by indigenous<br>communities in the southern Western Ghats of India	https://www.sysrev.com/u/14052/p/139927/article/96359371	http://dx.doi.org/10.1016/j.japb.2016.04.00
**RDNo.48**	Doc61. Local Knowledge Tapir Mexico_Laveriega-Nolasco_2016..pdf	Lavariega Nolasco, M. C., BRIONES-SALAS, M., MAZAS-TEODOCIO, A., & DURÁN-MEDINA, E. (2016). Ecology and local knowledge of the Baird's tapir (Tapirella bairdii) in the Sierra Madre de Oaxaca, Mexico. Integrative zoology, 11(5), 361-374.	https://www.sysrev.com/u/14052/p/139927/article/96359372	doi: 10.1111/1749-4877.12183

**RDNo.49**	Doc62. Local Knowledge Species Java_Partasasmita_2016..pdf	Partasasmita, R., Iskandar, J., & Malone, N. (2016). Karangwangi people's (South Cianjur, West Java, Indonesia) local knowledge of species, forest utilization and wildlife conservation. Biodiversitas Journal of Biological Diversity, 17(1).	https://www.sysrev.com/p/139927/article/96359373	DOI: 10.13057/biodiv/d170123
**RDNo.50**	Doc64. Game Mammal Role._Barboza_2016..pdf	Barboza, R. R. D., Lopes, S. F., Souto, W. M., Fernandes-Ferreira, H., & Alves, R. R. (2016). The role of game mammals as bushmeat in the Caatinga, northeast Brazil. Ecology and Society, 21(2).	https://www.sysrev.com/u/14052/p/139927/article/96359375	http://dx.doi.org/10.5751/ES-08358-21020
**RDNo.51**	Doc65. Tortoises Explotation should be TEK bottom up comun managed_Rahman_2015..pdf	Partasasmita, R., Iskandar, J., & Malone, N. (2016). Karangwangi people's (South Cianjur, West Java, Indonesia) local knowledge of species, forest utilization and wildlife conservation. Biodiversitas Journal of Biological Diversity, 17(1).	https://www.sysrev.com/u/14052/p/139927/article/96359373	DOI: 10.13057/biodiv/d170123
**RDNo.52**	Doc66. Evaluating LEK monitor hunting_Parry_2015..pdf	Parry, L., & Peres, C. A. (2015). Evaluating the use of local ecological knowledge to monitor hunted tropical-forest wildlife over large spatial scales. Ecology and Society, 20(3).	https://www.sysrev.com/u/14052/p/139927/article/96359377	. http://dx.doi.org/10.5751/ES-07601
**RDNo.53**	Doc69. Wildlife Use & Taboos Conservation_Bobo_2014..pdf	Bobo, K. S., Aghomo, F. F. M., & Ntumwel, B. C. (2015). Wildlife use and the role of taboos in the conservation of wildlife around the Nkwende Hills Forest Reserve; South-west Cameroon. Journal of ethnobiology and ethnomedicine, 11, 1-24.	https://www.sysrev.com/p/139927/article/96359379	https://link.springer.com/content/pdf/10.1186/1746-4269-11-2.pdf
**RDNo.54**	Doc71. Culture and subsistence of the Tongwe_Nakamura_2015..pdf	Pruetz, J. D. (2017). MAHALE CHIMPANZEES: 50 YEARS OF RESEARCH Edited by Michio Nakamura Kazuhiko Hosaka Noriko Itoh Koichiro Zamma Cambridge, United Kingdom: Cambridge University Press. 2015. 780 pp. ISBN 978–1-107–05231-4 (Hardcover).	https://www.sysrev.com/p/139927/article/96359381	https://doi.org/10.1002/ajpa.23293
**RDNo.55**	Doc72. Community Local Perspectives for Conservation Papua_Heist_2015..pdf	Van Heist, M., Liswanti, N., Boissière, M., Padmanaba, M., Basuki, I., & Sheil, D. (2015). Exploring local perspectives for conservation planning: A case study from a remote forest community in Indonesian Papua. Forests, 6(9), 3278-3303.	https://www.sysrev.com/p/139927/article/96359382	doi:10.3390/f6093278
**RDNo.56**	Doc73. Food & Medicine use Opossum_Barros_2014..pdf	Barros, F. B., & de Aguiar Azevedo, P. (2014). Common opossum (Didelphis marsupialis Linnaeus, 1758): food and medicine for people in the Amazon. Journal of Ethnobiology and Ethnomedicine, 10, 1-14.	https://www.sysrev.com/p/139927/article/96359383	https://ethnobiomed.biomedcentral.com/articles/10.1186/1746-4269-10-65
**RDNo.57**	Doc74. Quantit Analysis Paiwan TEK_Wu_2014..pdf	Wu, C. S., Wu, M. S., & Chen, Y. H. (2014). Quantitative Analysis of Traditional Ecological Knowledge: A Case Study of Paiwan People in Jialan Village, Taiwan. 台灣林業科學, 29(3), 205-219.	https://www.sysrev.com/p/139927/article/96359385	https://ws.tfri.gov.tw/001/Upload/OldFile/files/29_3_04Traditional%20Ecological%20Knowledge.pdf
**RDNo.58**	Doc75. Subsistence Hunting C.paca Brasil_Valsecchi_2014..pdf	Valsecchi, J., El Bizri, H. R., & Figueira, J. E. C. (2014). Subsistence hunting of Cuniculus pacain the middle of the Solimões River, Amazonas, Brazil. Brazilian Journal of Biology, 74, 560-568.	https://www.sysrev.com/p/139927/article/96359386	http://dx.doi.org/10.1590/bjb.2014.0098
**RDNo.59**	Doc81. Porcupine Local Knowledge and Threats_Castilho_2013..pdf	Castilho, L. C., Martinez, R. A., Giné, G. A., Ribeiro, G. C., & Schiavetti, A. (2013). The thin-spined porcupine, Chaetomys subspinosus (Rodentia: Erethizontidae), within protected areas in the Atlantic Forest, Brazil: local knowledge and threats. Tropical Conservation Science, 6(6), 796-810.	https://www.sysrev.com/p/139927/article/96359392	https://journals.sagepub.com/doi/pdf/10.1177/194008291300600607
**RDNo.60**	Doc83. Wildmeat or Bushmeat_SubsistenceHunt_Commer Harvst_PangauAdam_2012..pdf	Pangau-Adam, M., Noske, R., & Muehlenberg, M. (2012). Wildmeat or bushmeat? Subsistence hunting and commercial harvesting in Papua (West New Guinea), Indonesia. Human Ecology, 40, 611-621.	https://www.sysrev.com/p/139927/article/96359394	DOI 10.1007/s10745-012-9492-5
**RDNo.61**	Doc87. Use of Terrestrial Fauna Caicaras Brasil_Hanazaki_2009..pdf	Hanazaki, N., Alves, R. R., & Begossi, A. (2009). Hunting and use of terrestrial fauna used by Caiçaras from the Atlantic Forest coast (Brazil). Journal of Ethnobiology and Ethnomedicine, 5, 1-8.	https://www.sysrev.com/p/139927/article/96359398	doi:10.1186/1746-4269-5-36

**RDNo.62**	Doc68. Bushmeat & Zoonosis_Friant_2015.(1).pdf	Friant, S., Paige, S. B., & Goldberg, T. L. (2015). Drivers of bushmeat hunting and perceptions of zoonoses in Nigerian hunting communities. PLoS neglected tropical diseases, 9(5), e0003792.	https://www.sysrev.com/p/139927/article/98523959	doi:10.1371/journal.pntd.0003792
